# Evaluation of the role of kefir in management of non-alcoholic steatohepatitis rat model via modulation of NASH linked mRNA-miRNA panel

**DOI:** 10.1038/s41598-022-27353-x

**Published:** 2023-01-05

**Authors:** Noha Salah, Sanaa Eissa, Amal Mansour, Nagwa M. Abo El Magd, Amany Helmy Hasanin, Manal M. El Mahdy, Mohamed Kamel Hassan, Marwa Matboli

**Affiliations:** 1 Medical Biochemistry and Molecular Biology Department, Faculty of Medicine, Ain Shams University, Abbassia, P.O. box 11381, Cairo, Egypt; 2 MASRI institute of research, Faculty of Medicine, Ain Shams University, Cairo, Egypt; 3 Department of Medical Microbiology and Immunology, Faculty of Medicine, Ain Shams University, Cairo, Egypt; 4 Department of Clinical Pharmacology, Faculty of Medicine, Ain Shams University, Cairo, Egypt; 5 Department of Pathology, Faculty of Medicine, Ain Shams University, Cairo, Egypt; 6 Department of Biology, Faculty of Science, Port Said University, Port Said, Egypt; 7 Center for Genomics, Helmy Institute for Medical Science, Zewail City for Science & Technology, Giza, Egypt

**Keywords:** Molecular biology, Gastroenterology, Molecular medicine

## Abstract

Non-alcoholic steatohepatitis (NASH) is the clinically aggressive variant of non-alcoholic fatty liver disease. Hippo pathway dysregulation can contribute to NASH development and progression. The use of probiotics is effective in NASH management. Our aim is to investigate the efficacy of kefir Milk in NASH management via modulation of hepatic mRNA-miRNA based panel linked to NAFLD/NASH Hippo signaling and gut microbita regulated genes which was identified using bioinformatics tools. Firstly, we analyzed mRNAs (SOX11, SMAD4 and AMOTL2), and their epigenetic regulator (miR-6807) followed by validation of target effector proteins (TGFB1, IL6 and HepPar1). Molecular, biochemical, and histopathological, analyses were used to evaluate the effects of kefir on high sucrose high fat (HSHF) diet -induced NASH in rats. We found that administration of Kefir proved to prevent steatosis and development of the inflammatory component of NASH. Moreover, Kefir improved liver function and lipid panel. At the molecular level, kefir down-regulated the expression of miR 6807-5p with subsequent increase in the expression of SOX 11, AMOTL2 associated with downregulated SMAD4, resulting in reduction in the expression of the inflammatory and fibrotic markers, IL6 and TGF-β1 in the treated and prophylactic groups compared to the untreated rats. In conclusion, Kefir suppressed NASH progression and improved both fibrosis and hepatic inflammation. The produced effect was correlated with modulation of SOX11, SMAD4 and AMOTL2 mRNAs) – (miR-6807-5p) – (TGFB, IL6 and, HepPar1) expression.

## Introduction

Nonalcoholic fatty liver disease (NAFLD) is a pathological condition with accumulation of fat in the liver with no excessive alcohol intake, varying from simple hepatic steatosis (SS) to nonalcoholic steatohepatitis (NASH) with fibrosis and cirrhosis^1^. Due to the increasing epidemic of obesity and type 2 diabetes worldwide, a 178% rise in liver deaths has been estimated because of NASH by 2030^2^**.** NASH could be induced by multiple conditions acting in parallel, including genetic predisposition, oxidative stress, abnormal lipid metabolism, lipotoxicity, mitochondrial dysfunction, endoplasmic reticulum stress, altered production of cytokines and adipokines and gut dysbiosis. Accordingly, hepatic inflammation could precede steatosis in NASH^3^. At the present time, no FDA-approved medical treatment for NASH or liver fibrosis are confirmed^4^**.**

Hippo signaling pathway was previously reported to have a critical role in the regulation of hepatic size, proliferation, apoptosis, and stress response^5^. Accordingly, its dysregulation could have an effect on NASH pathogenesis via liver dedifferentiation and tumorigenesis^6^.

Moreover, former data have reported that noncoding RNAs (ncRNA), including microRNAs (miRNAs) and long noncoding RNAs (lncRNAs)^7^ may play crucial regulatory roles in NASH initiation and progression. The coregulatory links between these ncRNAs may demonstrate the molecular regulation and its involvement in NASH progression and could be used as appropriate biomarkers for assessing severity of the disease^8^**.**

Additionally, the use of intestinal microbiota as a potential therapeutic target for NASH have been achievable. Probiotics are live microorganisms, which give health benefits to the host if consumed in appropriate amounts^9^. Probiotics slow down disease progression and hinder gastrointestinal complications by affecting intestinal flora, intestinal permeability, and inflammatory response^10^. Currently, the use of probiotics in the management of NAFLD have not been addressed by any guidelines and the molecular mechanisms of their health benefits are not entirely known as well^11^.

Kefir is an acidic-alcoholic fermented milk, produced by kefir grains. Therefore, it is stable and has a precise balance of lactic acid bacteria and yeast^12^. It contains around 30 unique species of “good bacteria” that could benefit gut health^13^ and so used as a potential therapy to treat gastrointestinal diseases and ischemic heart disease^14^ because of its microflora, and the existence of some metabolites as organic acids. Further, it has several biological effects e.g. antibacterial, antioxidant, immunomodulatory, antidiabetic, and cholesterol lowering actions^15^. Ho et al. 21 reported that kefir milk has anti-adipogenic effects by suppressing adipocyte differentiation and inhibiting the expression of the SREBP-1, ACC and FAS proteins during cholesterol synthesis^16^.

Clearly, bioinformatics presents novel clues and initial data for screening potential biomarkers for different diseases and drug monitoring^17^. Moreover, it can be used to identify genes related to specific biological functions and predict drug-molecular signaling that may lead to a breakthrough in targeted therapy^18^.

Therefore, the present study aimed at investigating the efficacy of kefir Milk in NASH management via modulation of hepatic mRNA-miRNA based panel linked to NAFLD/NASH Hippo signaling and gut microbiota regulated genes which are identified using bioinformatics tools.

## Materials and methods

### Chemicals and drugs

Cholesterol and cholic acid were purchased from Ralin BV (Lijinbaan, Netherlands). Ready-made kefir milk was purchased from Heal Pharmaceutical every week and stored at temperature (4 °C), it contains *Lactobacillus lactis* as the largest bacterial colony representing approximately 80% and *Lactobacillus paracasei*, *Lactobacillus acidophilus*, *Lactobacillus delbrueckii ssp. bulgaricus*, *Lactiplantibacillus plantarum*, *Lactobacillus kefiranofaciens,* *Leuconostoc* *mesenteroides*, *Streptococcus thermophilus* and *yeast* of kefir (Danisco®) representing the other 20%.

The total number of micro-organisms in the fermented milk produced contain at least 10^7^ colony-forming units (CFU)/ml and the yeast number not less than 10^4^ CFU/ml^19,20^.

### Experimental animals and diets

Forty male (6 weeks-old) Wistar rats (140–160 g) were obtained from the Scientific Research Institute, Cairo, Egypt, housed under specific conditions (20 ± 2 °C), 12 h light/dark cycle with free access to water and normal rat chow. All experimental procedures were done according to the guidelines of the Institutional Animal Care and were approved by the Ethics Committee of Ain Shams Faculty of Medicine, Egypt (Ethical Approval Number; FWA000017585).

**Figure 1:**
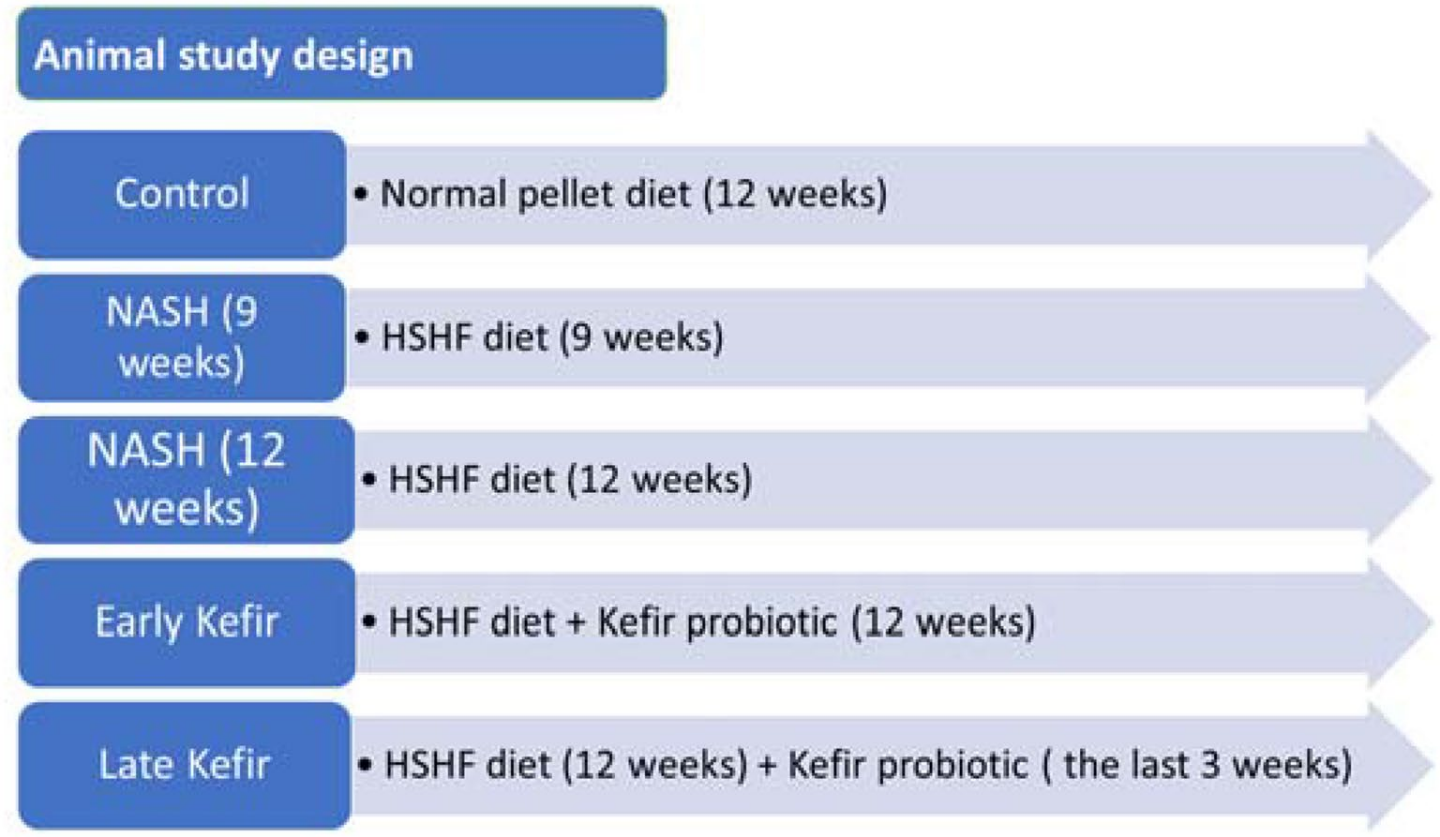
Flow chart showing the experimental design of the animal studies. *NASH* nonalcoholic steatohepatitis, *HSHF* high sucrose and high fat.

After a one-week acclimatization period, the rats were randomly divided into three groups (n = 8 for each group where treatment was given orally by gavage^21^: (I) Normal control group (NC) fed a normal pellet diet; (II) NASH model group, this group was further subdivided into IIa: (9-weeks NASH model) and IIb: (12-weeks NASH model)^22^, Group III (probiotic-treated), this group was further subdivided into IIIa: (NASH/early probiotic-treated), rats were fed HSFD for 12 weeks and received probiotic treatment daily from day one for 12 weeks and IIIb: (NASH/late probiotic-treated), rats were fed HSHF for 12 weeks and received probiotic treatment daily in the last 3 weeks. NASH model was induced by feeding rats high fat/high sucrose diet consisting of 70% standard chow, 20% lard, 10% sucrose, 1% cholesterol, and 0.25% cholic acid^22^. Kefir milk was mixed and then administered by oral gavage at a dose of 1.8 mL/rat/day. All rats were weighed at the beginning of the study, at the end of each week, and before animal sacrifice (Fig. 1).

### Blood sample and liver tissue collection

At the end of the study, rats were fasted for 12 h. and anesthetized with one dose of urethane (1.2 g/kg, IP)^23^ for sacrificing. Blood samples were collected from retro-orbital vein, centrifuged at 3000 rpm for 10 min for serum separation, then aliquoted and stored at − 20 °C for subsequent biochemical analysis. Whole livers were removed promptly, weighed and dissected. Part of the hepatic tissues was immediately stored in − 80 °C for RNAs and protein assessment whereas the remaining parts were rapidly fixed in 10% neutral buffered formalin for histopathological and immunohistochemical analyses.

### Assessment of liver function and lipid profile markers

We assessed levels of serum aspartate transaminase (AST), alanine transaminase (ALT), total and direct bilirubin, gamma-glutamyl transferase (GGT), alkaline phosphatase (ALP) and Lipid profile [Total cholesterol (TC), Triglycerides (TG), HDL cholesterol (HDL-C), and LDL cholesterol (LDL-C)] in serum samples^24^ using commercial kits according to the manufacturer’s instructions and proceeded by a multifunctional biochemistry analyzer (AU680, Beckman Coulter Inc, CA).

### Hepatic histopathological evaluation

For light microscopy, the buffered formalin-fixed liver tissues were dehydrated in ethanol, immersed in paraffin wax and sliced into Sections (5-μm thick). Standard hematoxylin–eosin (HE) for assessment of histological features of steatohepatitis and Masson’s Trichrome staining for detecting collagen fibers were performed^25^.

Each section was scored for the severity of hepatic steatosis, inflammation and fibrosis individually according to the published criteria^26^, Table 1.

**Table 1:** Histological grading of steatosis, inflammation and staging of fibrosis.

Steatosis	Grades	
	**0**	˂ 5% steatosis
	**1**	5–25% steatosis
	**2**	26–50% steatosis
	**3**	51–75% steatosis
	**4**	More than 75% steatosis
Inflammation	Grades	
	**0**	None
	**1**	Focal collection of mononuclear cells
	**2**	Diffuse infiltrates of mononuclear cells
	**3**	Focal collection of polymorphonuclear cells
	**4**	Diffuse infiltrates of polymorphonuclear
Fibrosis	Stages	
	**F0**	No evidence of fibrosis
	**F1**	Mild fibrosis
	**F2**	Moderate fibrosis
	**F3**	Advanced fibrosis

### In-silico filtration of hepatic mRNA-miRNA based panel linked to hippo signaling, gut microbita regulated genes and NAFLD/NASH

Firstly; we have selected AMOTL2, SOX11 and SMAD4 mRNAs from public microarray dataset; QuickGO (https://www.ebi.ac.uk/QuickGO/), Comparative Toxicogenomics Database (http://ctdbase.org/) and Gene atlas database (https://www.ebi.ac.uk/gxa) and by literature reviews because of their strong correlation to NASH pathogenesis (Supplementary Fig. 1A–E). Secondly; we verified the gene ontology of the selected mRNAs and their link to Hippo signaling and hepatocyte stem cell proliferation/differentiation using Harmonizome database (http://amp.pharm.mssm.edu/Harmonizome/) (Supplementary Fig. 2A–E). Thirdly; the selected mRNAs were verified for their relation to gut microbiota related genes by using Encyclopedia of gut microbiota regulated genes (http://microbiota.wall.gu.se/) (Supplementary Fig. 3A–C). Fourthly, AMOTL2, SOX11 and SMAD4 mRNAs were then imported into Protein–Protein Interaction Networks Functional Enrichment Analysis (STRING) database (https://string-db.org/) to ensure their protein protein interaction and their linkage with HIPPO pathway target effectors (Supplementary Fig. 4A). Then, the mRNA was mapped to Hippo signaling using KEGG pathway of hippo (Supplementary Fig. 4B). Fifthly, we used miRWalk 3.0 (http://mirwalk.umm.uni-heidelberg.de/), that combine the prediction results of both TargetScan and MiRBase to retrieve has-miR-6807-5p based on target complementarity and ranking score (Supplementary Fig. 5A–C). We verified the expression of has-miR-6807-5p in liver using miRmine database (Supplementary Fig. 6A). https://guanfiles.dcmb.med.umich.edu/mirmine/index.html). Lastly; pathway enrichment analysis of has-miR-6807-5p by using DIANA database tools: miRpath (http://snf-515788.vm.okeanos.grnet.gr/) that confirmed its relation to Hippo signaling and stem cell proliferation/differentiation (Supplementary Fig. 6B).

### Extraction of total RNA (mRNA and miRNA)

Total RNA from the liver tissue was extracted^27^ using miRNeasy Mini Kit (Cat. No. 217004, Qiagen, Germany) as per instructions from the manufacturer. The concentration and purity of total RNA were assessed using NanoDrop (Thermoscientific, USA); the purity of the isolated RNAs (A260/A280) was 1.8–2. The reverse transcription of the extracted total RNA into complementary DNA (cDNA) was immediately proceeded with miScript II RT (Cat. No. 218161, Qiagen) and RT2 First Strand Kit (Cat.No. 330404, Qiagen) according to the manufacturer’s protocol using Thermo Hybaid PCR express (Thermo Fisher Scientific, Massachusetts, USA).

### Real-time quantitative polymerase chain reaction (RT-qPCR)

The expression levels of SMAD4, SOX11 and AMOTL2 mRNAs ((Accession: NM_NM_005359.6, NM_ _003108.4 and NR_002819, _001113490.2, respectively)) in the liver tissues were assessed using a Quantitect SYBR Green ROX qPCR Mastermix Kit (Cat no. 330523) (Qiagen, Germany), hsa-miR-6807-5p (Accession: MIMAT0027514: 5' gugagccaguggaauggagagg 3′) expression in tissue samples was assessed by using miScript SYBR Green PCR Kit (Cat. No. 218073, Qiagen, Germany). The GAPDH and Hs_SNORD72_11 miScript Primer were used as the house keeping gene to normalize the raw data and then compared with a control sample. RT-qPCR amplification was performed in Applied Biosystems 7500 Real Time PCR system (Applied Biosystems, Foster City, USA) thermal cycler. The PCR program for the SYBR Green-based qPCR was as follows: denaturation at 95 °C for 15 min; 40 cycles of denaturation for 10 s at 94 °C; then annealing for 30 s at 55 °C; and lastly, extension for 30 s at 70 °C. Each reaction was performed in duplicate. The threshold cycle (Ct) value of each sample was calculated using The Applied Biosystems™ 7500 Real-Time PCR System version 2.0 software which also calculated the efficiency of the PCR (Applied Biosystems). The melting curves were analyzed to affirm the specificities of the amplicons for the SYBR Green-based PCR amplification. Calculation of the relative quantification of RNA expression was performed by the software according to Livak method, where RQ = 2^−ΔΔCt^^28,29^**.**

### Detection of Hepatocyte specific antigen (Hep Par 1) as Protein-based biomarkers for NAFLD/NASH progression by immunohistochemical staining^30^

Immunohistochemistry was performed on liver sections embedded in paraffin (4 μm) using Benchmark Ventana (GX) automated staining platform (Ventana Medical Systems, USA). Liver sections were put on positively charged slides, dewaxed in xylene, and rehydrated using graded alcohols. Where, heat-induced epitope retrieval was done with Ventana Cell Conditioning Solution 1 for 48 min and the diluted primary antibodies and Hepatocyte specific antigen (Hep Par 1) (EP265), monoclonal rabbit antibody (7 ml prediluted, dilution 1:100. Ref no: 264R-18, Lot no: 0000027465) were applied to sections at 37 °C for 32 min. The presence of antigen was visualized using Ventana ultraView Universal DAB Detection Kit. Slides were counterstained with hematoxylin, dehydrated, cleared, and mounted with a permanent mounting media. The result of immunohistochemistry staining was counted by using the method described by^31^ to calculate the histoscore (H-score) which involves a semiquantitative assessment of both the percentage of positive cells and the intensity of staining (graded as: 0, non-staining; 1, weak staining; 2, medium staining; and 3, strong staining). Hep Par 1 positive cells were counted using fraction area in Image J software.

### Quantification of hepatic inflammatory cytokine IL-6 and fibrotic marker TGF-β1 by Enzyme Linked Immunosorbant Assay (ELISA)

Interleukin-6 (IL-6) and transforming growth factor β1 (TGF-β1) in liver tissues were determined^32^ using sandwich ELISA kits (cat no: E0079r and E0124R, respectively; EIAab, Wuhan, China) and according to manufacturer’s instructions. IL-6 and TGF-β1 were selected as they are either highly involved in cell proliferation or strongly correlated to Hippo signaling pathway as well as strongly interacted with the pre-selected protein markers through string tool.

### Statistical analysis

The results were expressed as mean ± SEM. Statistical analysis was carried out using GraphPad Prism, software program, version 6.0. Inc., CA, USA. Statistical difference among groups was determined using one-way ANOVA followed by post hoc test for comparison between more than two groups of parametric data. P values < 0.05 were considered statistically significant.

### Ethical approval &Institutional Review Board Statement

The study is reported in accordance with ARRIVE guidelines. All experimental procedures were done according to the guidelines of the Institutional Animal Care and were approved by the Ethics Committee of Ain Shams Faculty of Medicine, Egypt (Ethical Approval Number; FWA000017585).

## Results

### The effect of Kefir on body weight and relative liver weight

There was a significant increase in the body weight of the rats fed the HSHF diet in NASH groups compared with rats fed with the normal pellet in normal controls (NC), (P < 0.05). As well as Early Kefir group showed a significant decrease in body weight and the ratio of liver weight to body weight than NASH (12 weeks) group (P < 0.05), Table 2.

**Table 2:** The effect of kefir milk on body weight and liver mass in different study groups.

		Control (n = 8)	NASH (9 weeks) (n = 8)	NASH (12 weeks) (n = 8)	Early kefir treatment (n = 12)	Late kefir treatment (n = 10)
		Mean ± SD				
Body weight (g)	Week 0	144.5 ± 17	158.5 ± 16.1	153.3 ± 6.79	161.2 ± 17.37	151.5 ± 15.31
	Week 9	240** ± **32.95	288.8 ± 30.09^€^	277.9 ± 16.2^€^	269.9 ± 27.78^€a^	301.7** ± **18.79
	Week12	263.8** ± **41.4		293.8 ± 16.3	299.5** ± **29.57	312.7 ± 12
Liver weight (g)		5.62 ± 1.06	10.63 ± 1.99*	12.31 ± 1.03*	10.67** ± **1.23	10.8** ± **1.687

Values are mean ± SD; n: number of animals = 8 rats/each group. One-Way ANOVA test followed by Tukey’s multiple comparison test.

*P < 0.001 compared to control group.

^#^P < 0.001 compared to NASH (9 weeks) group.

^δ^P < 0.001 compared to NASH (12 weeks) group.

^a^P < 0.001 compared to Late Kefir.

^€^P < 0.05 compared to control group.

**Figure 2:**
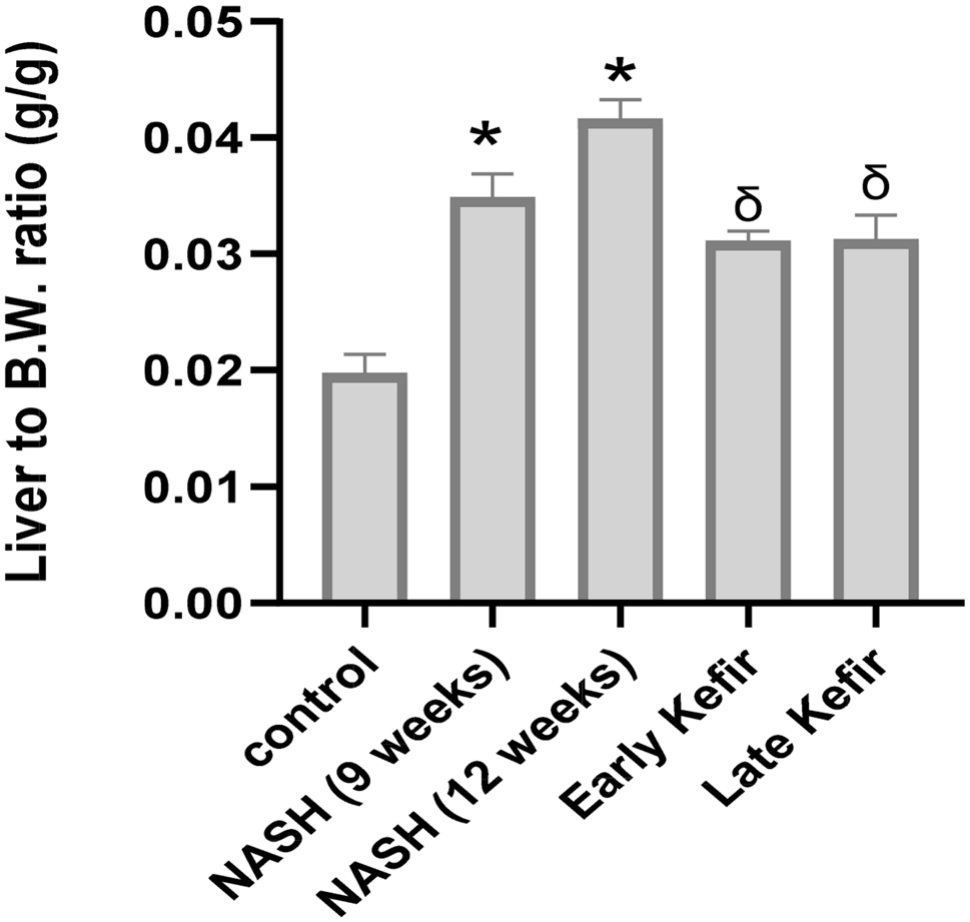
Liver/body ratio in different study groups. Values are mean ± SEM; number of animals = 8 rats/each group. One-Way ANOVA test followed by Tukey’s multiple comparison test. *P < 0.001 compared to control group, ^δ^P < 0.001 compared to NASH (12 weeks) group.

The previous results were also associated with a significant increase in liver relative weight in NASH groups (P < 0.001) compared with the NC Fig. 2. The liver to body weight ratio analysis revealed that both early and late Kefir treatment may be able to alleviate liver injury, where they showed significant decrease in the liver/body weight ratios (P < 0.001) compared to that of NASH group.

### Histopathological findings

The liver from NC rats had a visible normal brownish red color with smooth and shiny appearance. Whereas, NASH rats liver, showed the typical fatty liver features and looked enlarged and extensively infiltrated with yellowish spots. Additionally, a netlike pattern on the livers surface from NASH group revealed the existence of fibrosis. Liver tissue of HSHF-fed rats administered with either early or late Kefir treatment looked redder and lighter than those of NASH groups.

**Figure 3:**
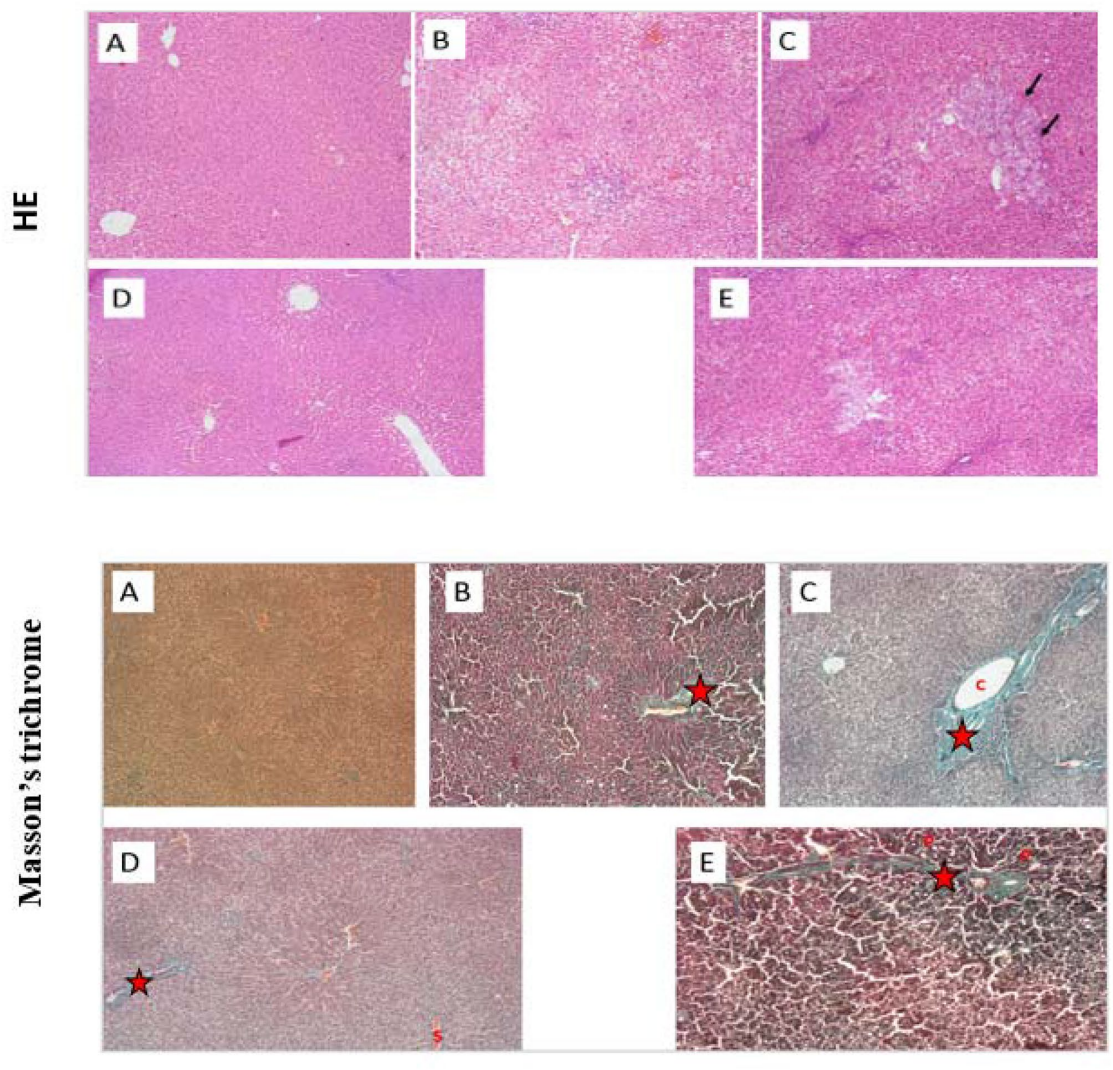
The effect of early and late Kefir treatment on hepatic steatosis, inflammation and fibrosis using HE and Masson’s trichrome staining (Magnifications: × 100). (**A**) Control group showed no pathological changes, (**B**) NASH (9 weeks) group developed mainly micro vesicular steatosis, mild hepatic lobular inflammation and mild fibrous septa(*star*) (**C**) NASH (12 weeks) group showed diffuse and extensive micro and macro-vesicular steatosis, severe lobular inflammation, focal necrosis, hepatocellular ballooning, marked expansion of facultative hepatic progenitor cells (*arrows*), dense fibrous septa and collagen fibers (*star*) (**D**) Early Kefir and (**E**) Late Kefir groups showed a lower degree of steatosis, inflammation and fibrosis, where Early Kefir showed more remarkable histological improvements.

Under the light microscope, liver of NC rats exhibited normal histological structure without inflammation or fibrosis. It was formed of classic hepatic lobules which were nearly hexagonal in shape. On contrary, NASH groups demonstrated development of steatosis with large fat droplets, inflammation and fibrosis were visible as well (Fig. 3). Compared with the NASH groups, the Kefir treated groups showed visible reductions in fat droplets, improvements in steatosis, and inflammation. The effects were more prominent in early treated group.

### The effect of Kefir treatment on liver function and lipid profile

As shown in Table 3**,** upon feeding the experimental rats with HSHF diet in NASH groups, the serum levels of AST, ALT, GGT, ALP, total bilirubin, and direct bilirubin have been increased significantly (P < 0.001), compared to the NC group. On contrary, oral administration of either early or late Kefir treatment , along with HSHF feeding, significantly reduced (P < 0.001) the elevated levels of these variables. Noticeably, this ameliorative effect was more remarkable when Kefir was administered prophylactically (Early Kefir).

**Table 3:** The effect of Kefir treatment on liver function and lipid profile.

Laboratory parameters	Control	NASH (9 weeks)	NASH (12 weeks)	Early kefir	Late kefir
	Mean ± SD				
AST (U/L)	20.38 ± 3.5	71.1 ± 4.6*	80.5 ± 5.451* ^#^	33.67 ± 5.48^# δ a^	60.8** ± **4.367^#δ^
ALT (U/L)	27 ± 4.5	81.88 ± 3.94*	126.6 ± 20.03*^#^	41 ± 4.936^#δa^	59.6** ± **3.34^δ#^
GGT (U/L)	14.38 ± 2.2	64 ± 7.03*	79.38 ± 13.39*^#^	32.83 ± 5.02^#δa€^	43.3 ± 9.62^δ#^
ALP (U/L)	29.13 ± 7.7	90.75 ± 3.15*	105.9 ± 7.2*^#^	51.5 ± 6.11^#δa^	68.4 ± 7.57^#δ^
Total bilirubin (mg/dL)	0.308 ± 0.05	1.06 ± 0.17*	1.844 ± 0.505*^#^	0.463 ± 0.05^#δ^	0.73** ± **0.067^δ#€^
Direct bilirubin (mg/dL)	0.122 ± 0.02	0.57 ± 0.08*	0.806 ± 0.14*^#^	0.223 ± 0.05^#δ^	0.297** ± **0.0194^#δ^
Total cholesterol (mg/dL)	88.25 ± 7.7	121.1 ± 5.51*	181.9 ± 3.48*^#^	99 ± 9.323^#δa^	126.7 ± 1.63^δ^
Triglyceride (mg/dL)	45.38 ± 4.5	115.6 ± 5.9*	141.5 ± 10.06*^#^	73.5 ± 3.7^#δa^	94.5 ± 4.882^#δ^
HDL-C (mg/dL)	64.50 ± 5.8	32 ± 3.928*	30.13 ± 2.35*	50.17 ± 3.04^#δa^	60.5 ± 2.321^#δ^
LDL-C (mg/dL)	14.68 ± 4	65.9 ± 6.35*	123.5 ± 1.46*^#^	34.13 ± 11.3^#δa^	47.3 ± 4.187^#δ^

Values are mean ± SD; number of animals = 8 rats/each group. One-Way ANOVA test followed by Tukey’s multiple comparison test.

*LDL-C* low-density lipoproteins, *HDL-C* high-density lipoprotein, *AST* Aspartate aminotransferase, *ALT* Alanine aminotransferase, *ALP* Alkaline phosphatase, *GGT* Gamma-glutamyl transferase.

*P < 0.001 compared to control group.

^#^P < 0.001 compared to NASH (9 weeks) group.

^δ^P < 0.001 compared to NASH (12 weeks) group.

^a^P < 0.001 compared to Late Kefir.

^€^P < 0.05 compared to control group.

Regarding lipid profile, there was a significant (P < 0.001) upsurge in the serum levels of TC, TG, and LDL-C coupled with a significant (P < 0.001) decrease of serum HDL-C level in animals of NASH groups compared to NC group. NASH (12 weeks) group exhibited statistically significant increase than NASH (9 weeks) group regarding TC, Triglyceride, LDL, Liver function tests and TGFB1(P < 0.001).

When Kefir was given therapeutically simultaneously with HSHF diet, serum TC, TG and LDL-C levels decreased significantly (P < 0.001) while serum HDL-C increased significantly (P < 0.001), compared to NASH groups. These meliorative effects were augmented by prophylactic supplementation with Kefir. The achieved results showed that the use of gut microbiota-based treatments could decrease hepatocyte injury and improve serum lipid profile in NASH animal model.

### The effect of Kefir treatment on the expression of hepatic SOX11, SMAD4 and AMOTL2 mRNAs

**Figure 4:**
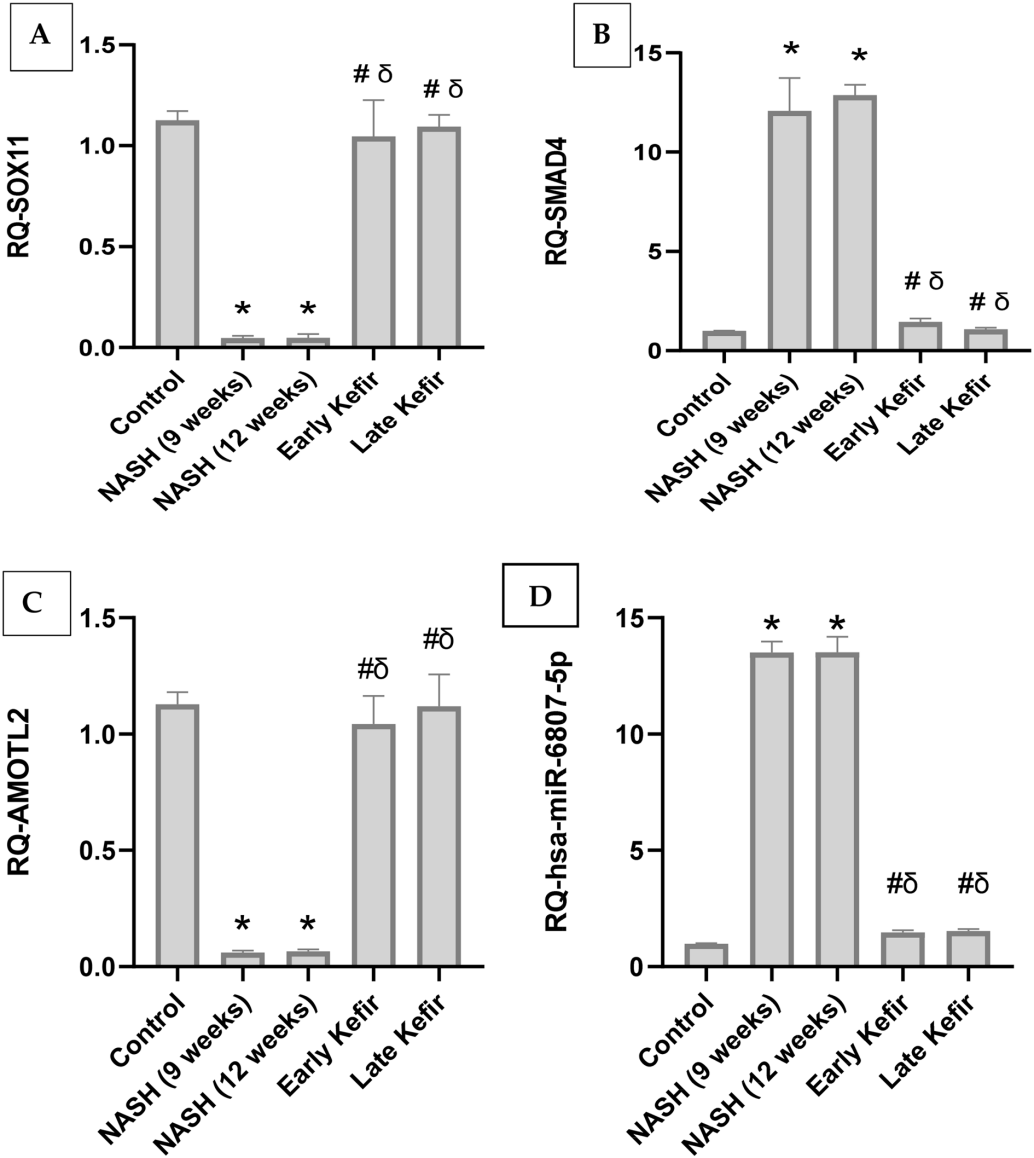
Effect of Kefir on the expression level of hepatic m-RNAs SOX11, SMAD4 and AMOTL2 (**A − C**), hepatic miR 6807-5p (**D**). Values are mean ± SEM; number of animals = 8 rats/each group. *P < 0.001 compared to control group, ^#^P < 0.001 compared to NASH (9 weeks) group, ^δ^P < 0.001 compared to NASH (12 weeks) group. One-way ANOVA followed by Tukey’s multiple comparison test. *RQ* relative quantification, *SOX11* SRY-Related HMG-box gene 11, *SMAD4* SMAD family member 4, *AMOTL2* Angiomotin-like 2, *hsa-miR 6807-5p* homo sapiens miR 6807-5p.

SOX11, SMAD4 and AMOTL2 expression levels were assessed in the liver tissue of the experimental animals (Fig. 4A − C). Results revealed that feeding rats with HSHF diet resulted in a sharp significant (P < 0.001) decrease in SOX11 expression level in NASH group animals. Kefir administration has normalized the significant decreases in hepatic SOX11 expression level shown in untreated NASH group rats. Additionally, no significant change in hepatic SOX11 expression level was observed between early and late treated groups. Meanwhile, NASH group recorded a remarkable significant increase in level of SMAD4(P < 0.001), compared to NC which was significantly corrected with Kefir treatment, compared to NASH group animals. Moreover, data manifested that hepatic AMOTL2 expression level in NASH group animals was significantly decreased (P < 0.001), in comparison to NC and was significantly corrected by Kefir administration.

### The effect of kefir treatment on the expression of hepatic miR-6807-5p

As shown in Fig. 4D, the RQ of miR-6807-5p in the liver tissue of NASH group animals was drastically increased (P < 0.001) compared to NC. Furthermore, both early and late Kefir treatment normalized the expression of hepatic miR-6807-5p, compared with that of NASH groups.

### The effect of kefir on hepatic cell differentiation and proliferation

**Figure 5:**
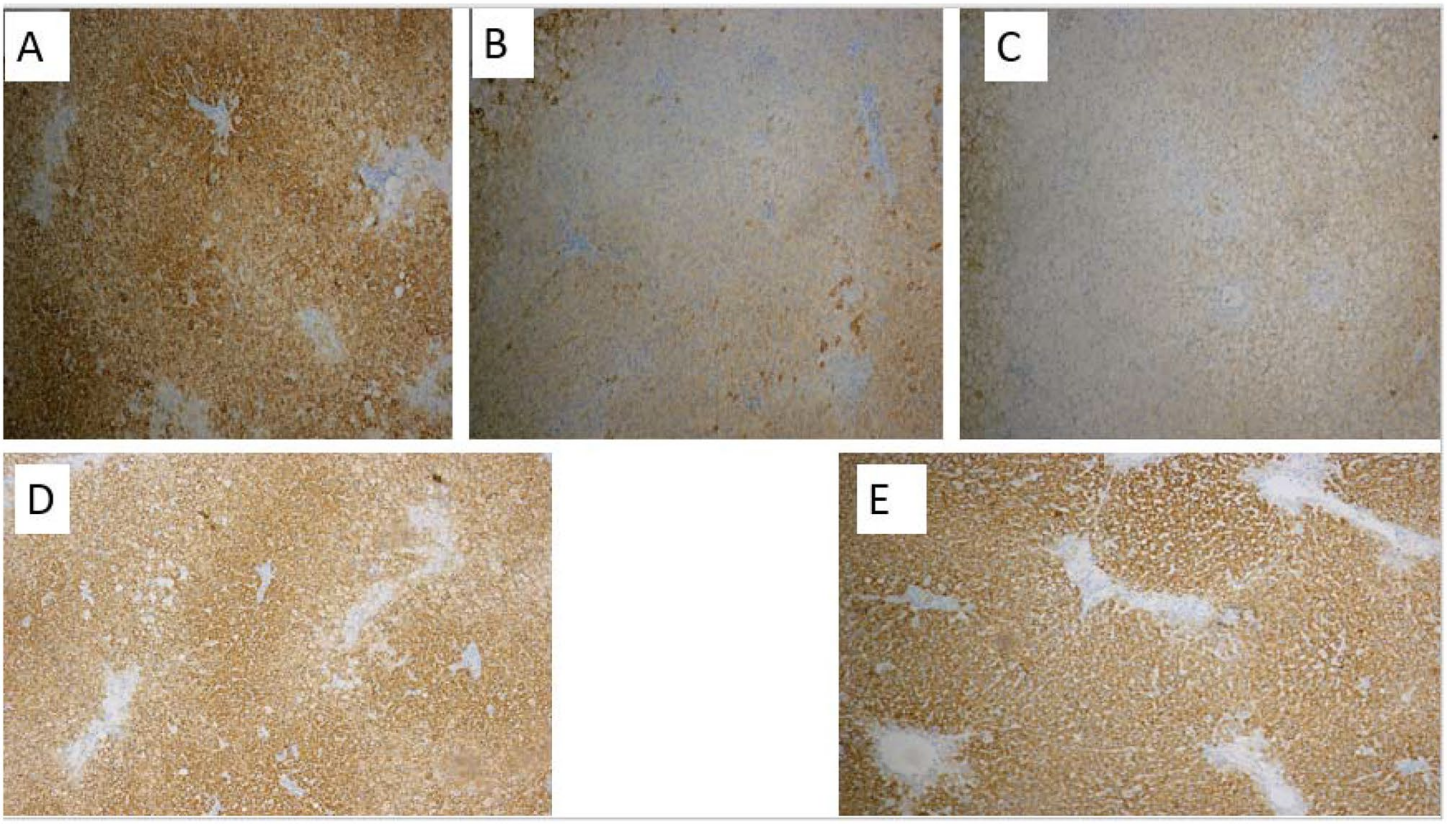
Immunohistochemical staining for hepatocyte specific antigen (HepPar1). (**A**) Controls showed a strong signal with Hep Par 1 (hepatocyte marker) producing distinct granular, cytoplasmic staining of hepatocytes, whereas in both (**B**) NASH (9 weeks) and (**C**) NASH (12 weeks) groups were mostly negative with HepPar-1. By comparison, (96%) hepatocytes in (**D**) Early Kefir group were immunoreactive for HepPar-1 and (**E**) Late Kefir group tended to show weaker, patchy positivity (HepPar1 × 100) indicated to hepatocyte differentiation.

**Figure 6:**
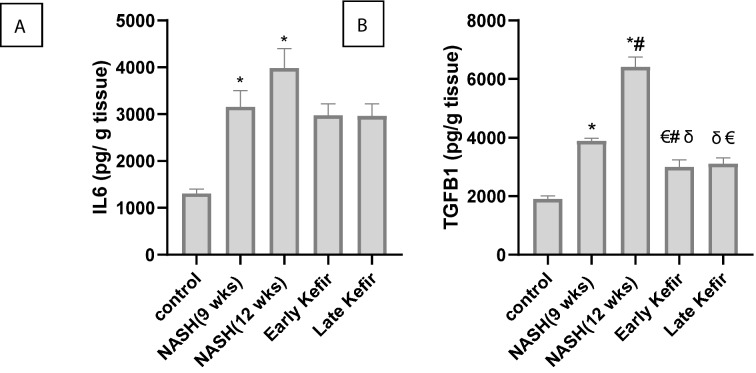
The effect of Kefir treatment on (**A**) Level of IL-6 (**B**) Level of TGF-β1 in liver tissues from various groups. Values are mean ± SEM, number of animals = 8 rats/each group. *P < 0.001 compared to control group, ^#^P < 0.001 compared to NASH (9 weeks) group, ^δ^P < 0.001 compared to NASH (12 weeks) group, ^€^P < 0.05 compared to control group. One-way ANOVA followed by Tukey’s multiple comparison test. *IL6* Interleukin 6, *TGFB1* transforming growth factor beta 1.

The state of liver progenitor cell proliferation and differentiation were evaluated in the liver by immunohistochemical analysis and assessment of Hepatocyte specific antigen (HepPar1). The increased in HepPar1 expression seen in NC (Fig. 6) compared to NASH groups results from Granular cytoplasmic staining due to mitochondrial binding. Hepatic progenitor cells (HPCs) gradually lose their biliary features, including markers as (HepPar1) and these transitional cells in the hepatocytic lineage become negative for the marker (Fig. 5B,C). Kefir administration showed up-regulation of HepPar1-positive cells in the liver tissues and the effect was more prominent in group received early treatment (Fig. 5D,E).

### The effect of Kefir on hepatic level of IL-6 and TGF-β1

The hepatic contents of IL-6 and TGF-β1 proteins were used as markers of inflammation and fibrogenesis, respectively. In the NASH groups, IL-6 and TGF-β1 proteins were significantly increased (P < 0.001, Fig. 6), compared to NC group. Kefir administration significantly decreased TGF-β1 compared with NASH groups (P < 0.001). These findings implied that both of early and late Kefir treatment effectively diminished the level of inflammation and fibrosis observed in NASH rats.

## Discussion

Non-alcoholic fatty liver disease (NAFLD) has become a very common disease because of the prevailing increase in obesity worldwide. Currently, several NAFLD therapies are being targeted to improve insulin resistance (IR), but there is no effective treatment^33^**.** Probiotic treatment was shown to improve NASH through modulating insulin resistance, the key factor which plays a major role in the development of a serious liver condition^34^**.** Due to correlation between small intestinal bacterial overgrowth (SIBO) and NAFLD observed in experimental and clinical studies^35,36^**,** probiotics could also delay disease progression and prevent complications by modulating intestinal flora, intestinal permeability, and inflammatory response^37^**.**

In this context, a high fat/high sucrose diet (HFD) induced NASH model experiment was conducted to assess the beneficial effects of the Kefir formula on the degree of hepatic fibrosis and steatosis, inflammation, and body composition via modulation at both epigenetic and genetic level. Previous studies have showed that kefir improved NAFLD regarding to BW, energy expenditure and basal metabolic rate through inhibition of the lipogenesis pathway^38^**.** This may explain our findings where the supplementation with Kefir prophylactically or therapeutically along with HFD, decreased the percentage and stage of steatosis, inflammation and fibrosis seen in NASH rats. Moreover, the serum levels of ALT and AST, and the hepatic content of fat, TGFB 1 and IL 6 proteins were significantly reduced with the treatment of the Kefir formula compared to NASH groups. In agreement with the previous finding that Kefir can diminish the inflammatory response in association with a decrease in tumor necrosis factor alpha (TNF-α), interleukin-1 beta (IL-1β) and transforming growth factor beta (TGF-β) cytokines^39^**.**

Hepatic stem/progenitor cells (HPCs or HpSCs, in humans) or oval cells (in rodents) are bipotential stem cells that can differentiate into mature hepatocytes and cholangiocytes^40^**.** They represent a reserve compartment that can be activated to reactive ductulus (Or ductular reaction: DR) only when the mature epithelial cells of the liver are continuously damaged or blocked in their replication or in cases of serious cell loss^41^**.**

Hippo pathway could promote cell death and differentiation and inhibit cell proliferation, however, the regulatory mechanisms for this signaling pathway are not clearly understood^42^. The downstream effector YAP1 is shown to be involved in hepatic cell proliferation, survival, development and differentiation^43^. Activated YAP1 leads to activation of hepatic stellate cells (HSC), prolonged activation of these cells causes liver fibrogenesis^44^**.** Therefore, a key objective is to understand the mechanisms that stimulate the switch of quiescent HSCs in a healthy liver to activated, myofibroblastic HSCs in NASH.

In this sense, variety of public microarray databases and computation algorithms have been investigated in the current work, for the selection of hepatic mRNA-miRNA panel linked to NAFLD/NASH Hippo signaling pathway and gut microbita regulated genes . We identified 3 mRNAs (SOX11, SMAD4 and AMOTL2), their epigenetic regulator (miR-6807) and their target effector proteins (TGFB, IL6 and HepPar1). Protein -protein interaction between the selected mRNAs protein products and HIPPO target effectors was obvious by STRING database as shown in (Supplementary Fig. 4A).

Cytosolic AMOTL2 proteins can attach YAP1 and TAZ in their unphosphorylated states, providing a Hippo independent mechanism to down-regulate the activities of these proteins^45^. AMOTL2 mRNA is downregulated in the current study in the rat liver tissue of NASH compared to NC or treated groups (P < 0.001), which indicated the freeing of YAP1 with subsequent activation of hepatic stellate cells and fibrogenesis participating in the pathogenesis of NASH. On the other hand, AMOTL2 is up regulated by the intake of kefir in rats which possibly ameliorates the features of NASH in the treated rat group by bounding YAP1.

SMAD4 interacts with SMAD2/3 and participates in the intracellular TGF-β signaling pathway. Knockout of SMAD4 from mesangial cells resulted in inhibition of TGF-β1-produced ECM synthesis^46^**.**These previous published results confirmed that SMAD4 has a significant role in the pathogenesis of fibrosis by controlling the ability of SMAD3 to activate transcription of a number of fibrogenic genes (collagens) , markers (α-SMA and E-cadherin)^47^ and explain the significant overexpression of SMAD4 in NASH group than NC and treated groups observed in the current study (P < 0.001).

SOX-11(SRY-related HMG-box) is a member of the group C SOX transcription factor family involved in the regulation of embryonic development and in the determination of the cell fate^48^. Sox 11 participates in positive regulation of hippo pathway leading to phosphorylation of YAP/TAZ, resulting in their cytoplasmic retention (Supplementary Fig. 2E). This may explain the decreased expression of its mRNA in NASH versus other study groups included in the current study (P < 0.001), with subsequent increase in nuclear translocation of YAP/TAZ forming functional transcriptional complexes with TEA domain proteins 1–4 (TEAD1–4)^49^. YAP/TAZ–TEADs promote the expression of Hippo-responsive genes that have a role in the production of proinflammatory cytokines (including interleukin 6 and TGF-β) and development of nonalcoholic steatohepatitis (NASH)^50^**.**

This agrees with another study that validated hepatic expression of a group of mRNAs and reported SOX11 downregulation in Obesity-Related Nonalcoholic Steatohepatitis compared to normal tissue^51^.

MiRNAs are shown to play important role in the development of diseases, including NASH^5^**.** In patients and animal models with NASH, circulating miRNAs represent significant differences compared to healthy controls**,** moreover, NASH shows significantly distinct miRNA expression profile compared to NAFLD^52^.

MiR 6807-5p is shown to be a novel microRNA biomarker for detecting gastric cancer (GC) but no previous reports have correlated its expression with the incidence or progression of NASH or with other liver diseases^53^. Our bioinformatics data analysis revealed its expression in liver (Supplementary Fig. 6A) and its correlation to Hippo signaling pathway (Supplementary Fig. 6B). Moreover, SOX11, SMAD4 and AMOTL2 are shown to be direct mRNA targets of miRNA-6807-5p as was retrieved from miRwalk database (Supplementary Fig. 5A–C). Our results revealed that there was a significant increase in miR 6807-5p concomitant with marked increase in SMAD4 and significant down regulation of SOX11, and AMOTL2 mRNA levels in the liver tissue of NASH group compared to NC or treated groups. This deregulated molecular profile reverts to normal after treatment with kefir milk.

TGF‐β was significantly increased in NASH as compared with other groups (P < 0.001) which is explained by previous work reporting that activation of TGF‐β signaling pathway was involved in the occurrence and development of NASH through motivating HSCs and the formation of extracellular matrix (ECM)^54,55^.

Continuous increased triacylglycerols buildup produces reactive oxygen species (ROS) and proinflammatory cytokines e.g. IL6, which induces NASH. IL-6 expression was significantly increased in the hepatic biopsy of patients with nonalcoholic steatohepatitis (NASH) than patients having simple steatosis or normal biopsies. Morever, there was a positive correlation between fibrosis staging and inflammation degree confirming the presence of hepatic IL-6 expression in human NASH^56^. Our results revealed a marked increase in IL-6 level in rat NASH liver when compared to NC (P < 0.001), but it was returned back to normal levels after kefir treatment.

In the present study, proliferation of oval cells was enhanced in NASH groups and decreased in Kefir groups. Previous studies found that HPC activation and the expansion of DR have been correlated with progressive fibrosis in adult and pediatric NASH and in HCV associated cirrhosis^57^**.**

In addition, upon differentiation towards hepatocytes, HPCs were previously shown to gradually lose their biliary features, including markers such as keratin 19 (K19) and keratin 7 (K7)^58^ as well as Hepatocyte Paraffin 1(Hep Par1). For the best of our knowledge, Hep Par1 was used for the first time as a marker of hepatocellular differentiation in NASH where it is shown to be mostly negative in the liver of NASH group while 96% of hepatocytes were immunoreactive for HepPar-1. with kefir treatment (Fig. 5).

**Figure 7:**
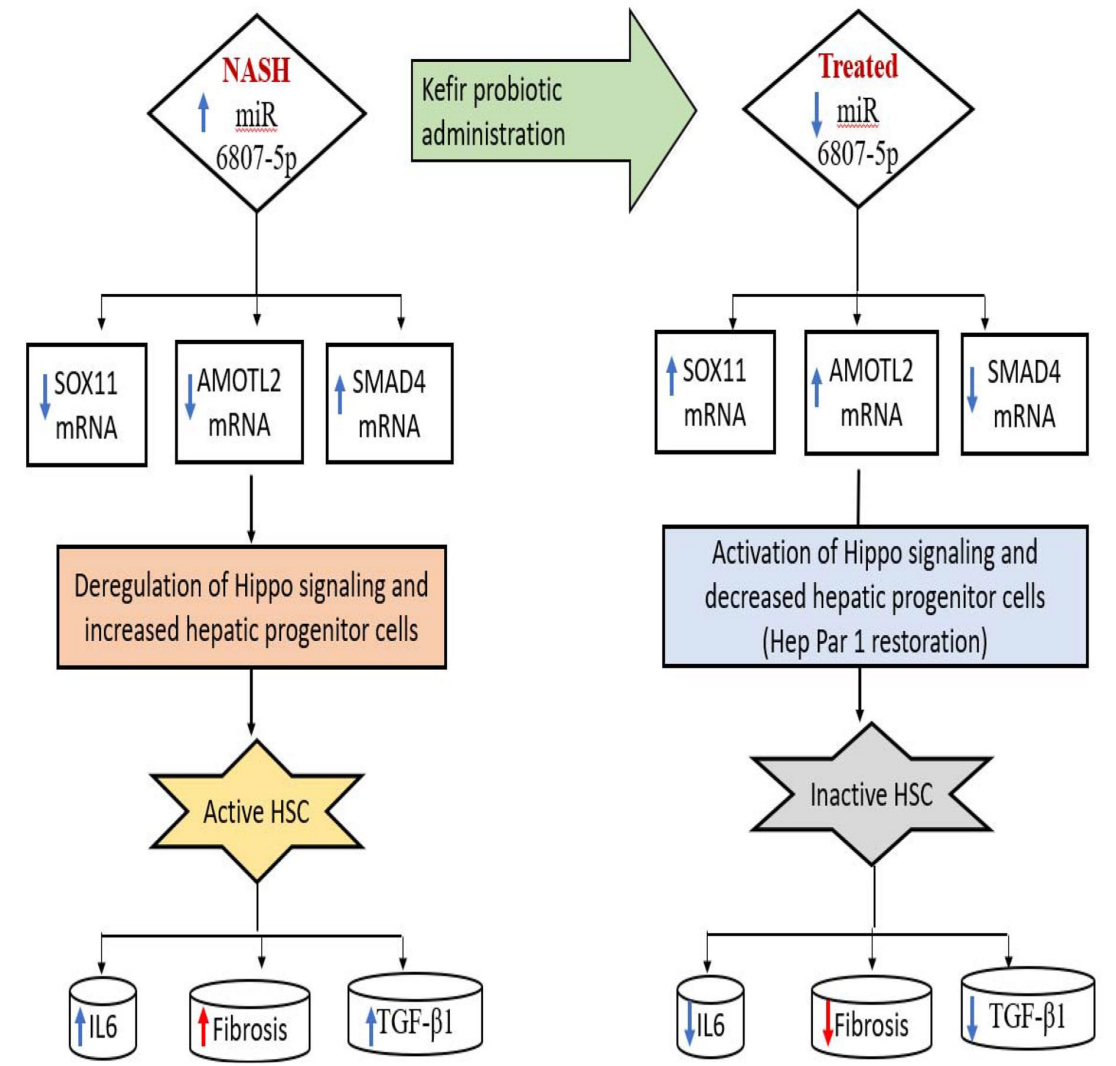
Proof of concept map of the study hypothesis.

Taken all together, our experimental model hypothesized that treatment with probiotic Kefir down-regulated miR 6807-5p with subsequent upregulation of SOX 11, and AMOTL2 and downregulation of SMAD4. Accordingly, YAP1 is negatively regulated with its subsequent sequestration in the cytosol. This cytoplasmic translocation of YAP1 inhibited cell proliferation and suppressed the expression of inflammatory IL6 and fibrotic TGF-β1 (Fig. 7).

## Conclusion

Kefir Milk was effective to improve the pathological and biochemical disturbances induced in NASH animal model through modulation of Hippo signaling/gut regulated genes linked RNA panel. This treatment down-regulated the expression of miR 6807-5p and SMAD4 as well as up-regulated the expression of SOX11& AMOTL2 mRNAs in rat hepatic tissue. The protein products of these genes interact with YAP1 in the Hippo signaling pathway controlling inflammation, and fibrosis progression in NASH.

## Limitations

Limitations of the present study includes the lack of Control rat group receiving only Kefir to show the intervention effect on miRNA-mRNA signature in a healthy model of rats. In addition, investigating the effect of kefir on the gut microbiota composition should be done in further studies.

## Supplementary Information

Supplementary Figures.

## Data Availability

Please contact corresponding author for data requests.

## Abbreviations

HSHF diet: High sucrose high fat diet
NAFLD: Nonalcoholic fatty liver disease
NASH: Non-alcoholic steatohepatitis
H-score: Histoscore
IL6: Interleukin 6
TGFb1: Transforming growth factor beta
Hep Par 1: Hepatocyte specific antigen
lncRNA: Long noncoding rna
miRNA: Micro-RNA
