# Supplementary material for: Evaluation of the role of kefir in management of non-alcoholic steatohepatitis rat model via modulation of NASH linked mRNA-miRNA panel
Source: Sci Rep. 2023 Jan 5;13:236. doi: 10.1038/s41598-022-27353-x (PMC9816104; doi:10.1038/s41598-022-27353-x)
Supplement: Supplementary file 1 — Supplementary Figures. [file 41598_2022_27353_MOESM1_ESM.pdf]

Supplementary Materials:

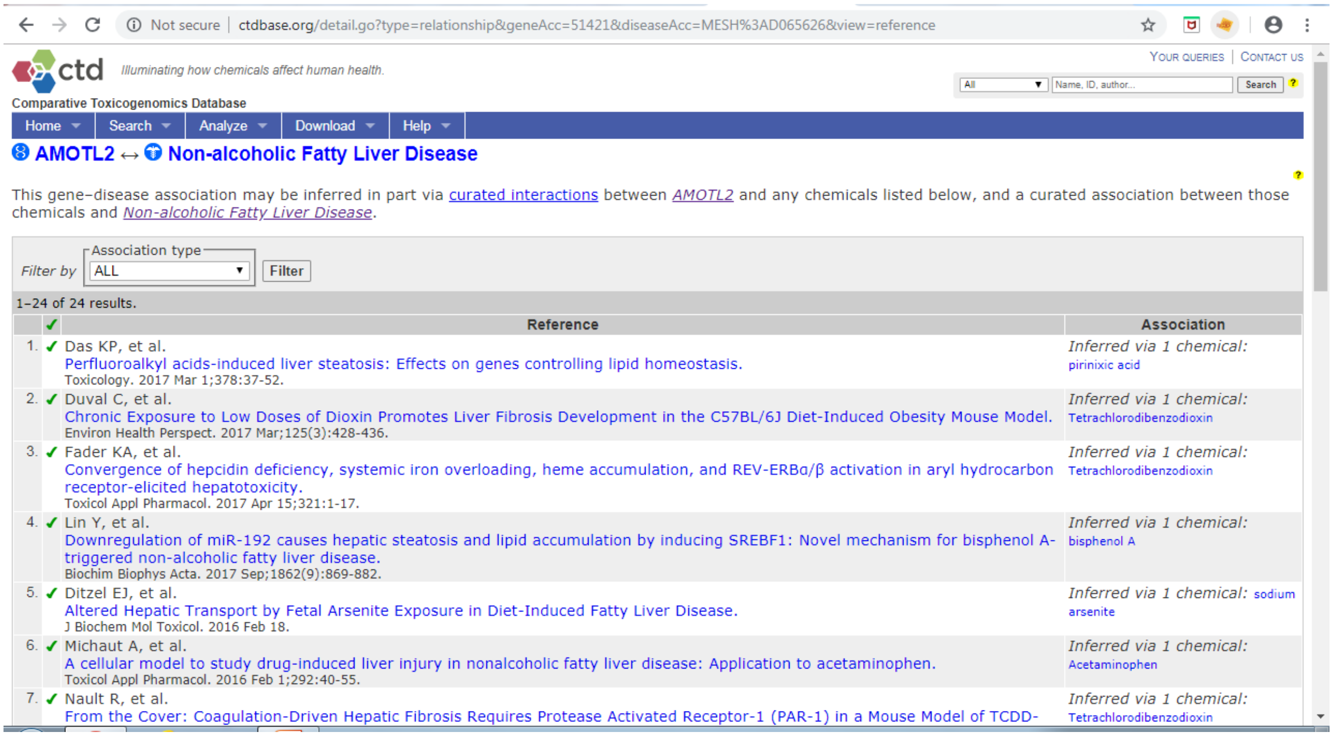

Figure S1A: Print screen shows correlation between AMOTL2 and NAFLD/NASH using Comparative Toxicogenomics Database,
http://ctdbase.org/detail.go?type=relationship&geneAcc=51421&diseaseAcc=MESH%3AD065626&view=reference

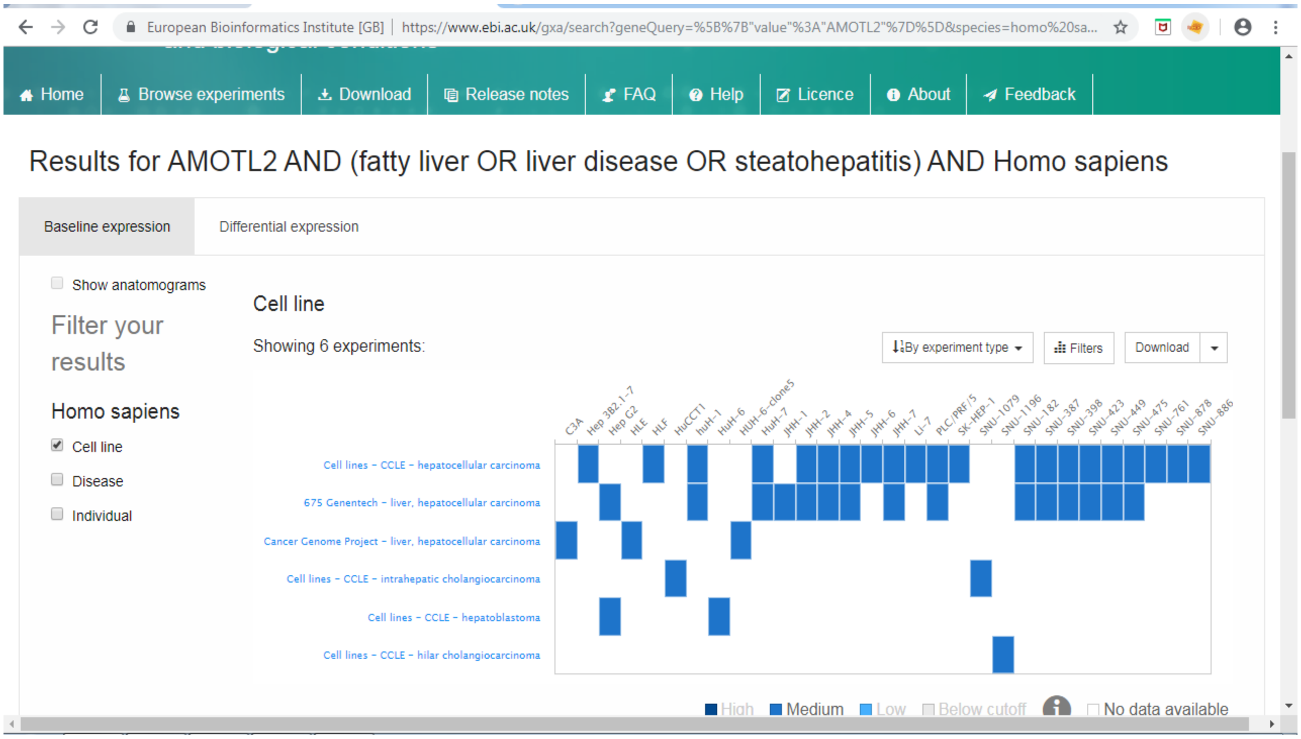

Figure S1B: Print screen shows correlation between AMOTL2 and NAFLD/NASH using Gene atlas database, https://www.ebi.ac.uk/gxa/search?geneQuery=%5B%7B%22value%22%3A%22AMOTL2%22%7D%5D&species=homo%20sapiens&conditionQuery=%5B%7B%22value%22%3A%22fatty%20liver%22%7D%2C%7B%22value%22%3A%22liver%20disease%22%7D%2C%7B%22value%22%3A%22steatohepatitis%22%7D%5D&bs=%7B%22homo%20sapiens%22%3A%5B%22CELL_LINE%22%5D%7D&ds=%7B%22kingdom%22%3A%5B%22animals%22%5D%7D#baseline

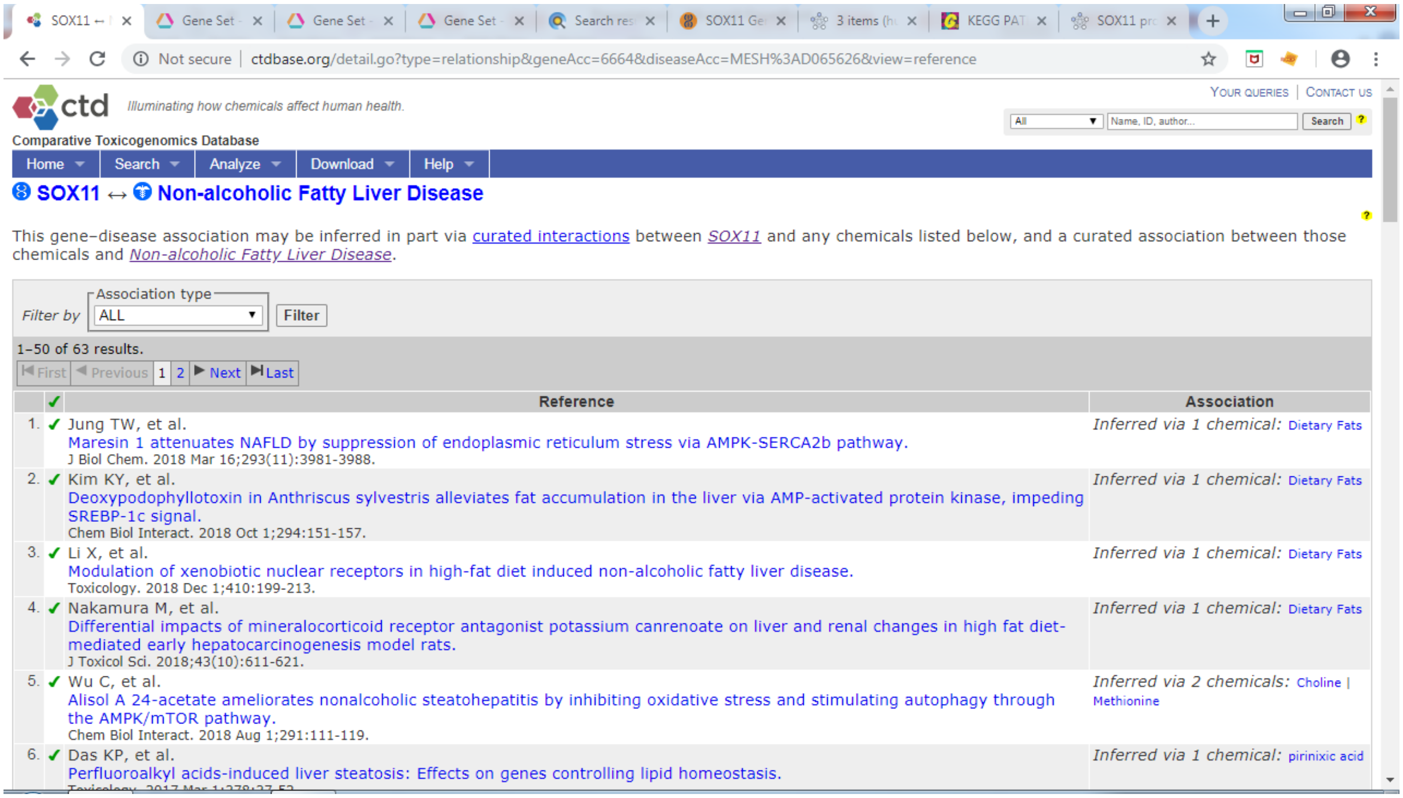

Figure S1C :Print screen shows correlation between SOX11 and NAFLD/NASH using Comparative Toxicogenomics Database,
http://ctdbase.org/detail.go?type=relationship&geneAcc=6664&diseaseAcc=MESH%3AD065626&view=reference

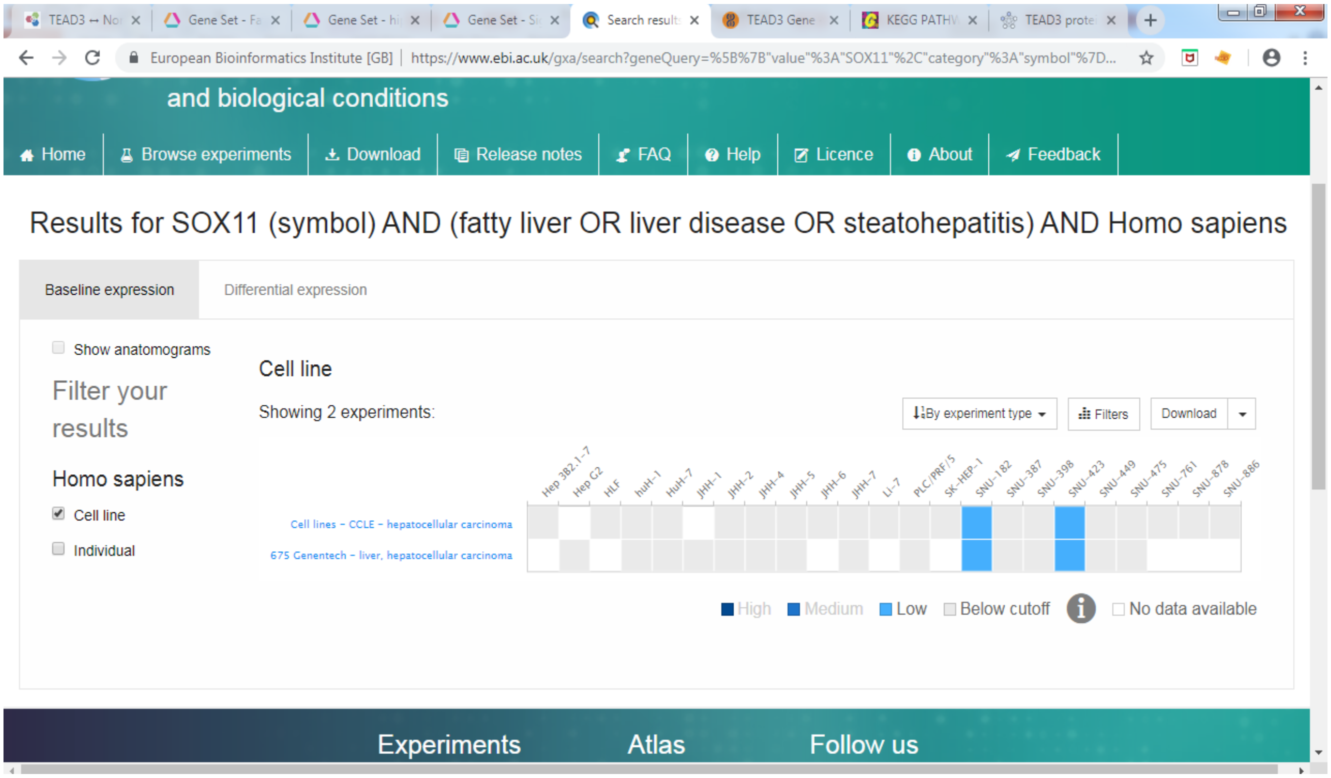

Figure S1D:Print screen shows correlation between SOX11 and NAFLD/NASH using Gene atlas database, https://www.ebi.ac.uk/gxa/search?geneQuery=%5B%7B%22value%22%3A%22SOX11%22%2C%22category%22%3A%22symbol%22%7D%5D&species=homo%20sapiens&conditio Query=%5B%7B%22value%22%3A%22fatty%20liver%22%7D%2C%7B%22value%22%3A%22liver%20disease%22%7D%2C%7B%22value%22%3A%22steatohepatitis%22%7D%5D&bs=%7B%22homo%20sapiens%22%3A%5B%22CELL_LINE%22%5D%7D#baseline

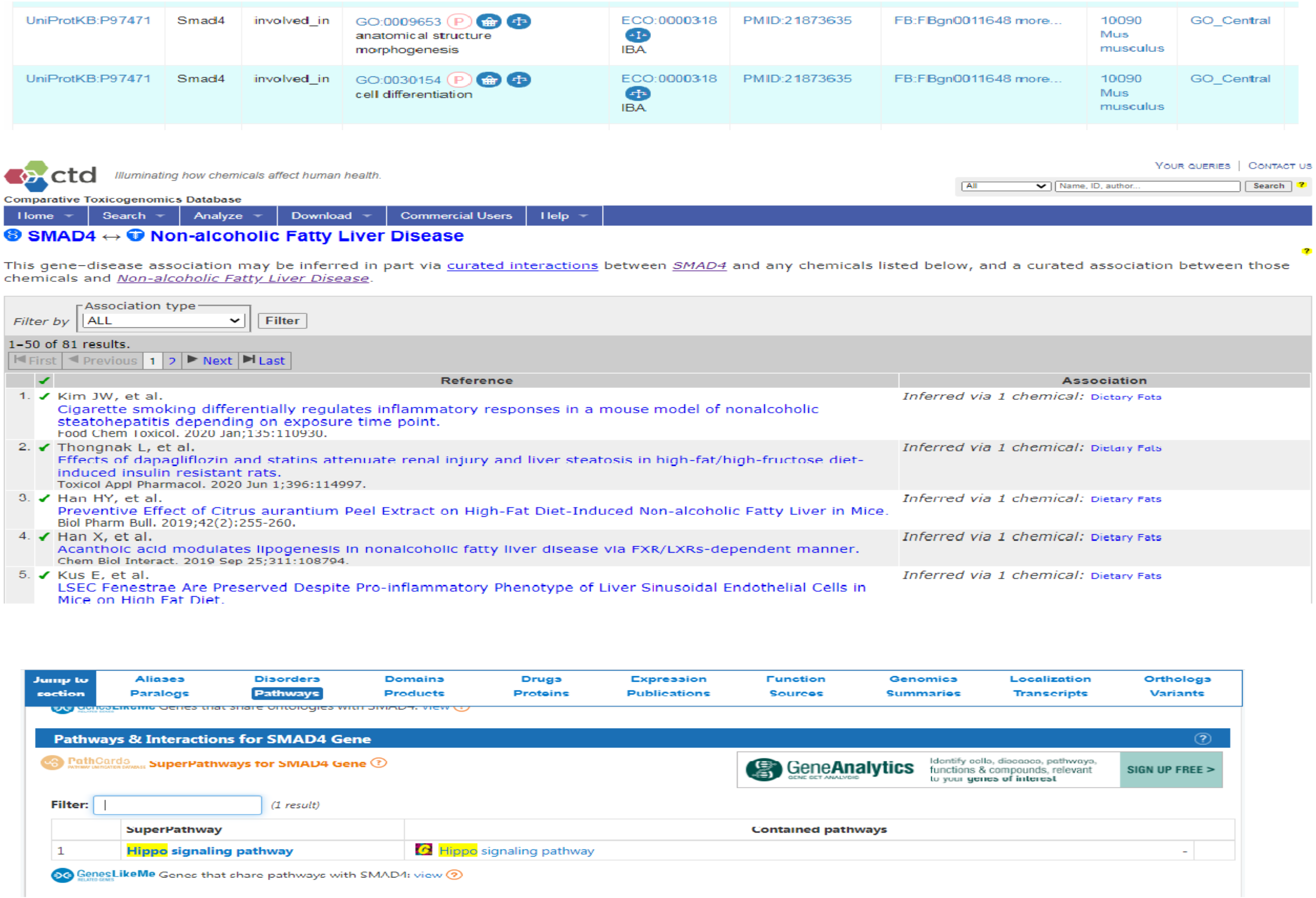

Figure S1E: Print screen shows correlation between SMAD4 and NAFLD/NASH using Comparative Toxicogenomics Database,

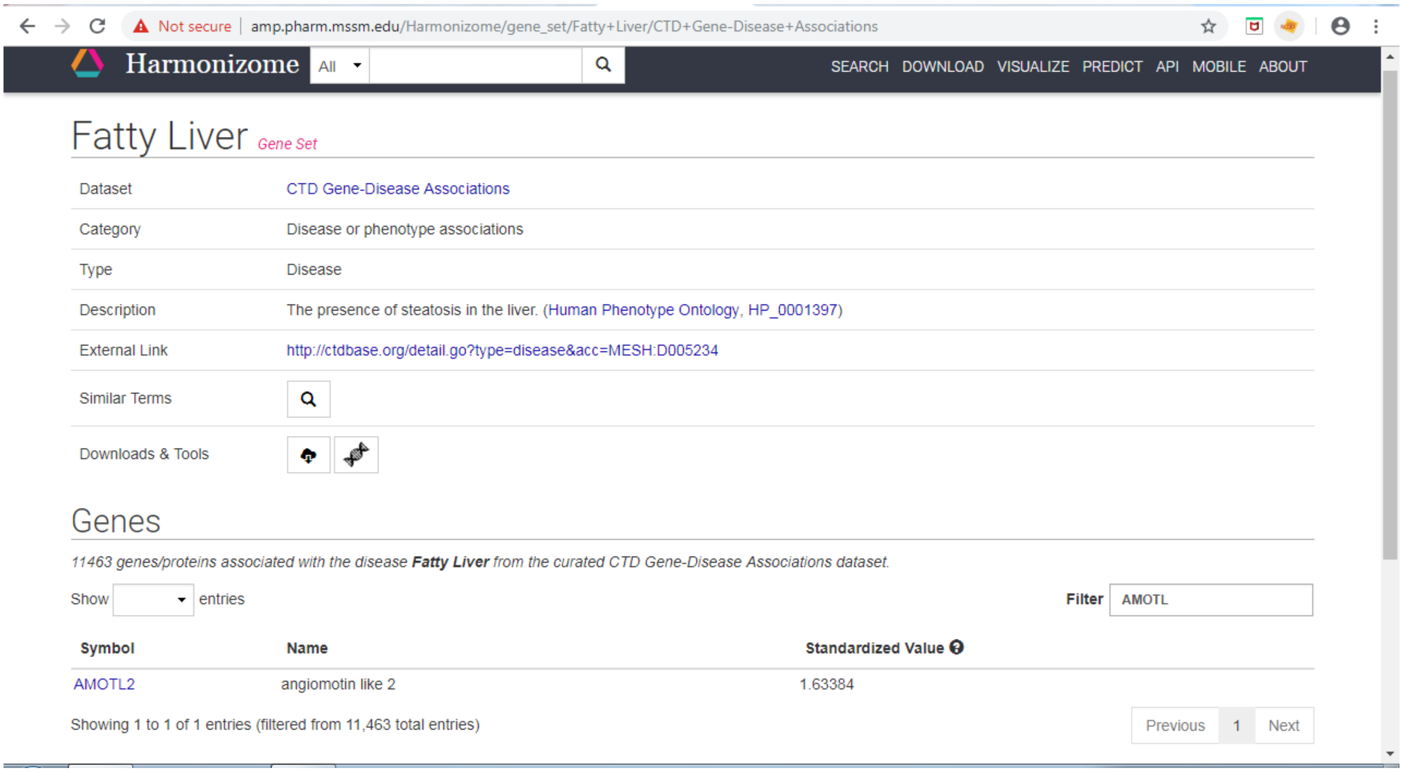

Figure S2A; Print screen shows correlation between AMOTL2 and Fatty liver using Harmonizome database: http://amp.pharm.mssm.edu/Harmonizome/gene_set/Fatty+Liver/CTD+Gene-Disease+Associations

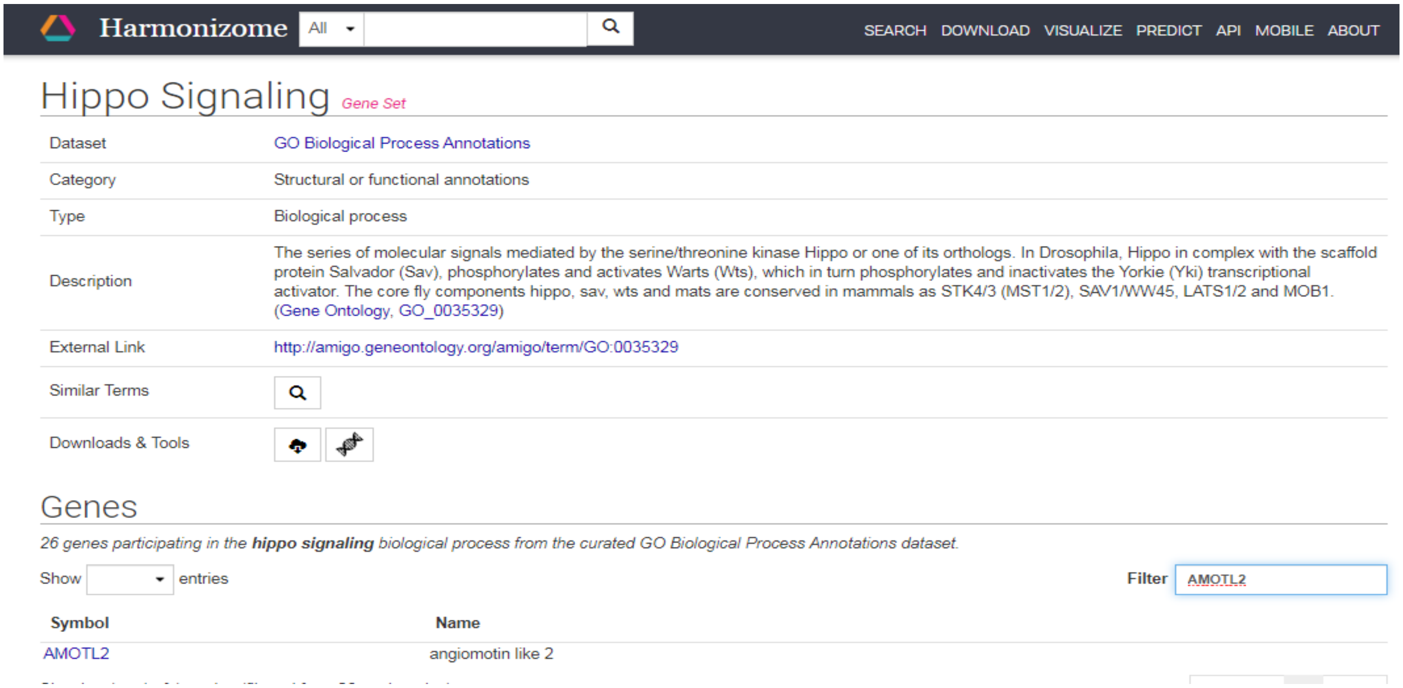

Figure S2B; Print screen shows correlation between AMOTL2 and Hippo signaling using Harmonizome

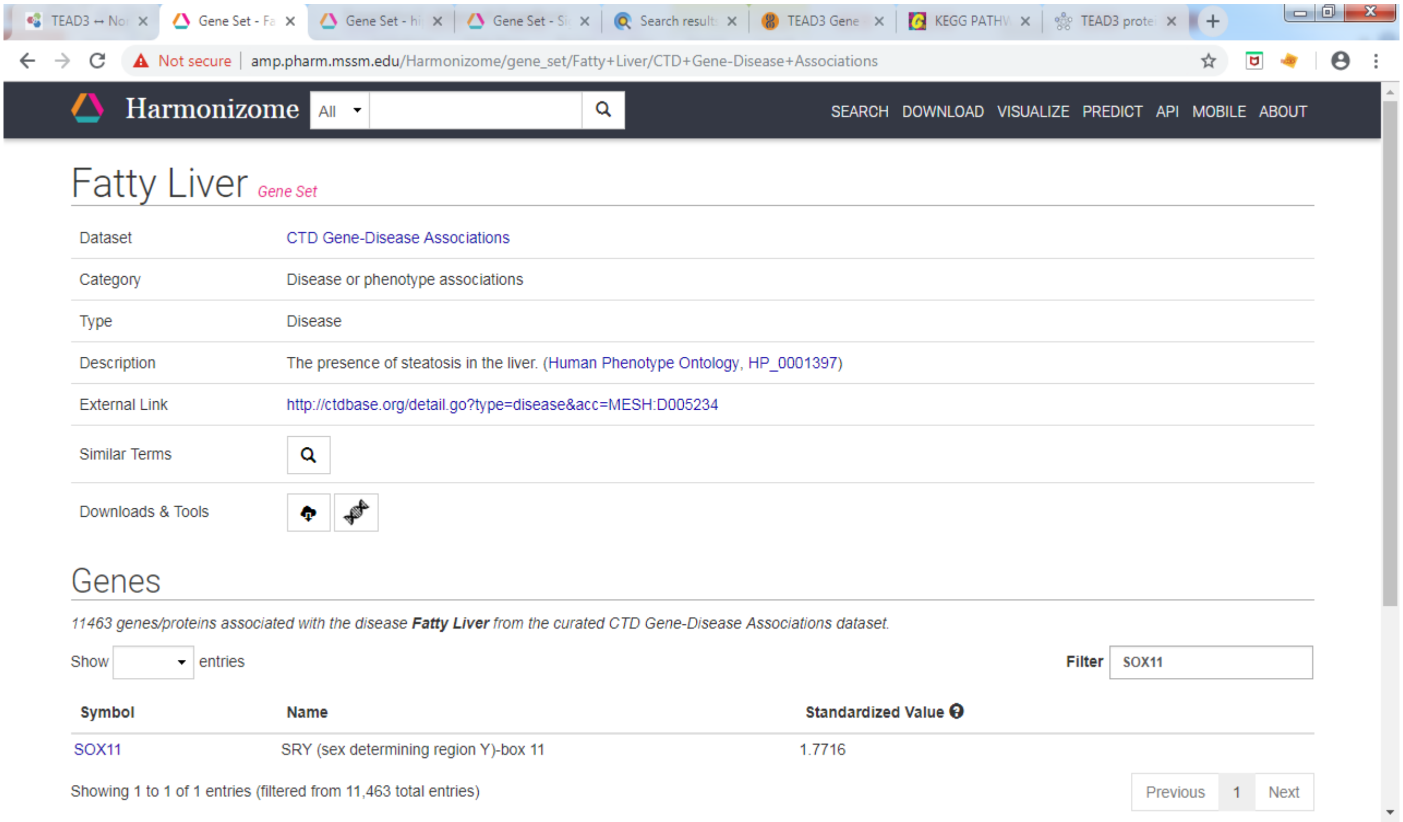

Figure S2C; Print screen shows correlation between SOX11 and Fatty liver using Harmonizome, http://amp.pharm.mssm.edu/Harmonizome/gene_set/Fatty+Liver/CTD+Gene-Disease+Associations

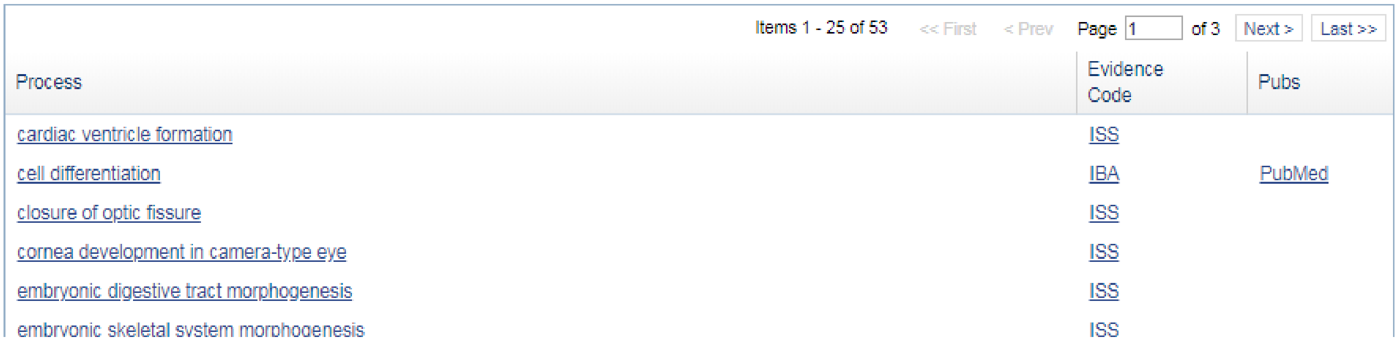

Figure S2D: Print screen shows correlation between SOX11 and Hippo signaling using ncbi Gene, https://www.ncbi.nlm.nih.gov/gene/6664

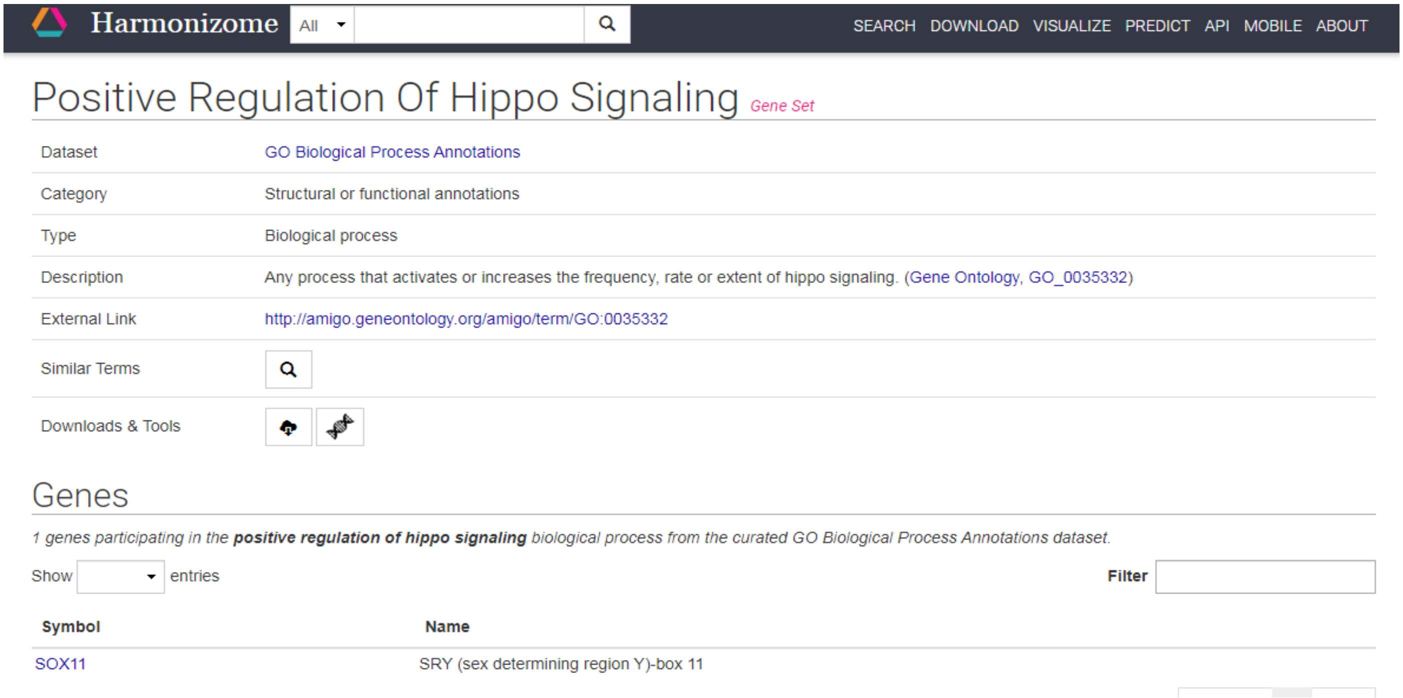

Figure S2E: Print screen shows correlation between SOX11 and Hippo signaling using Harmonizome, http://amp.pharm.mssm.edu/Harmonizome/gene_set/positive+regulation+of+hippo+signaling/GO+Biological+Process+Annotations

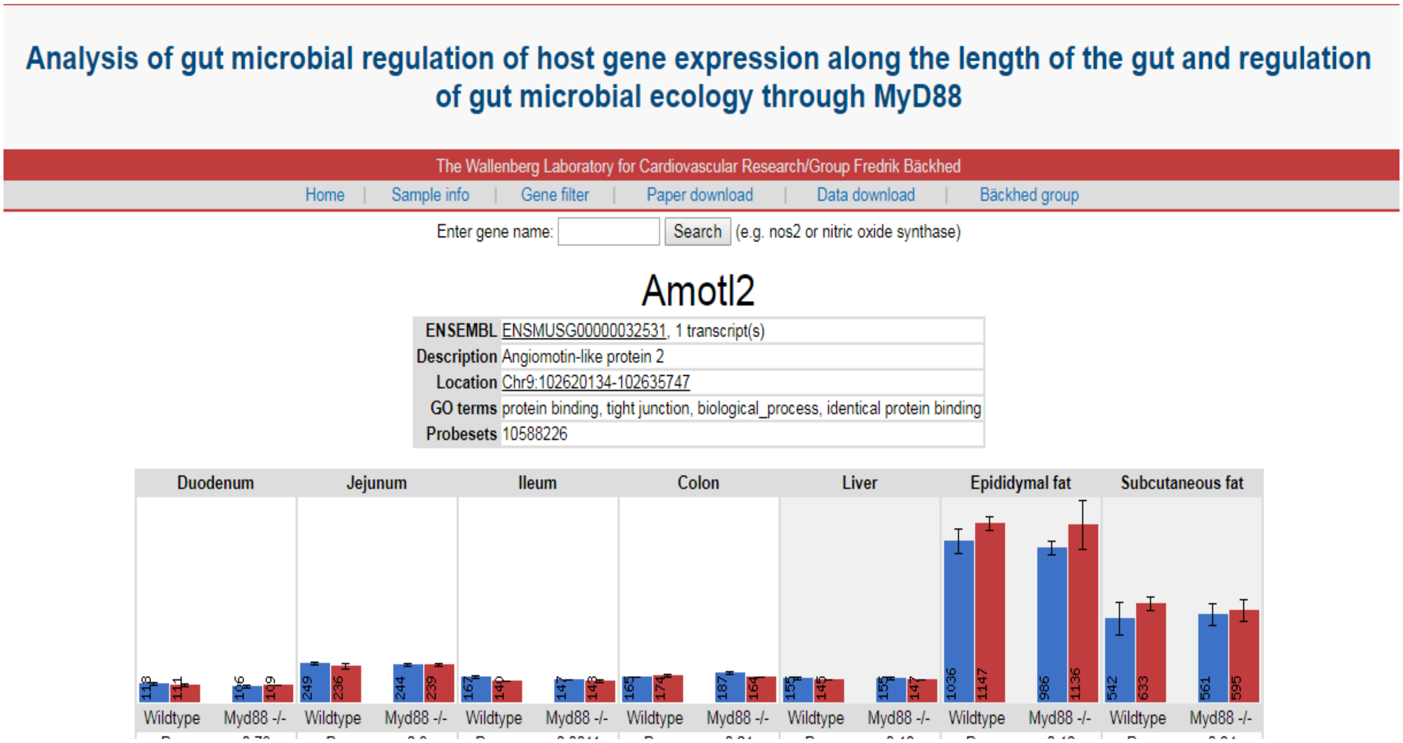

Figure S3A: Print screen shows Gut microbiota/ AMOTL2 crosstalk using Encyclopedia of gut microbiota regulated genes, [http://microbiota.wall.gu.se/larsson/view_gene.php?gene=8913.](http://microbiota.wall.gu.se/larsson/view_gene.php?gene=8913)

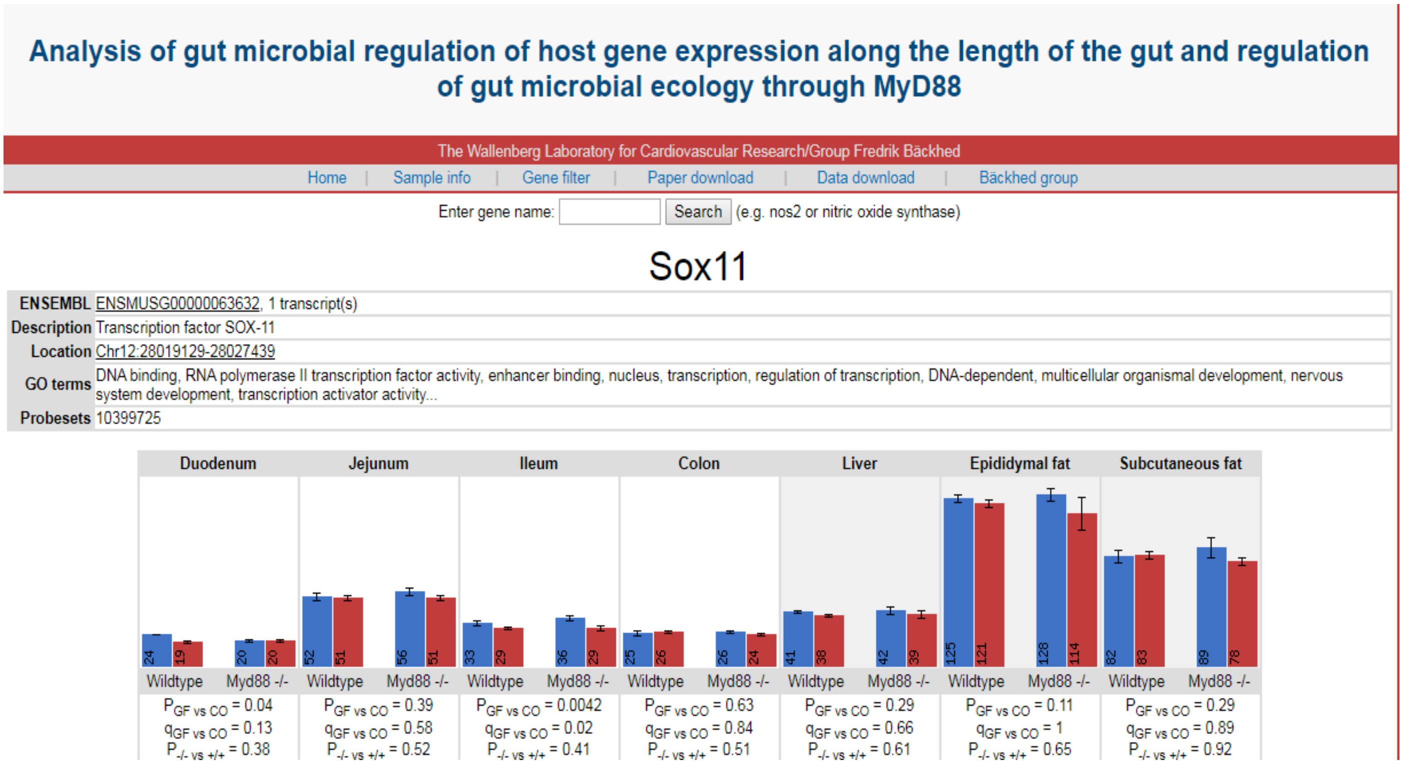

Figure S3B: Print screen shows Gut microbiota/ SOX11 crosstalk using Encyclopedia of gut microbiota regulated genes, http://microbiota.wall.gu.se/larsson/view_gene.php?gene=17626

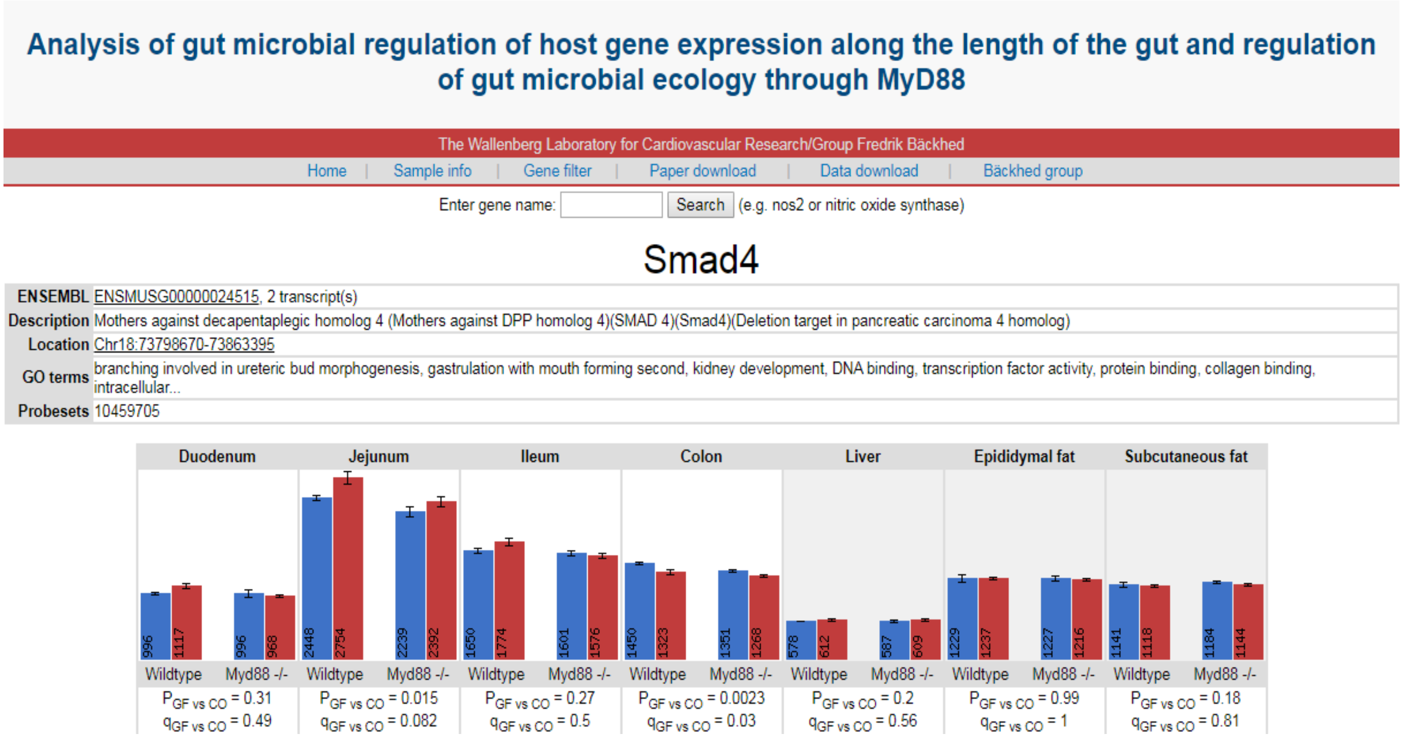

Figure S3C: Print screen shows Gut microbiota/ SMAD4 crosstalk using Encyclopedia of gut microbiota regulated genes, http://microbiota.wall.gu.se/larsson/view_gene.php?gene=4475

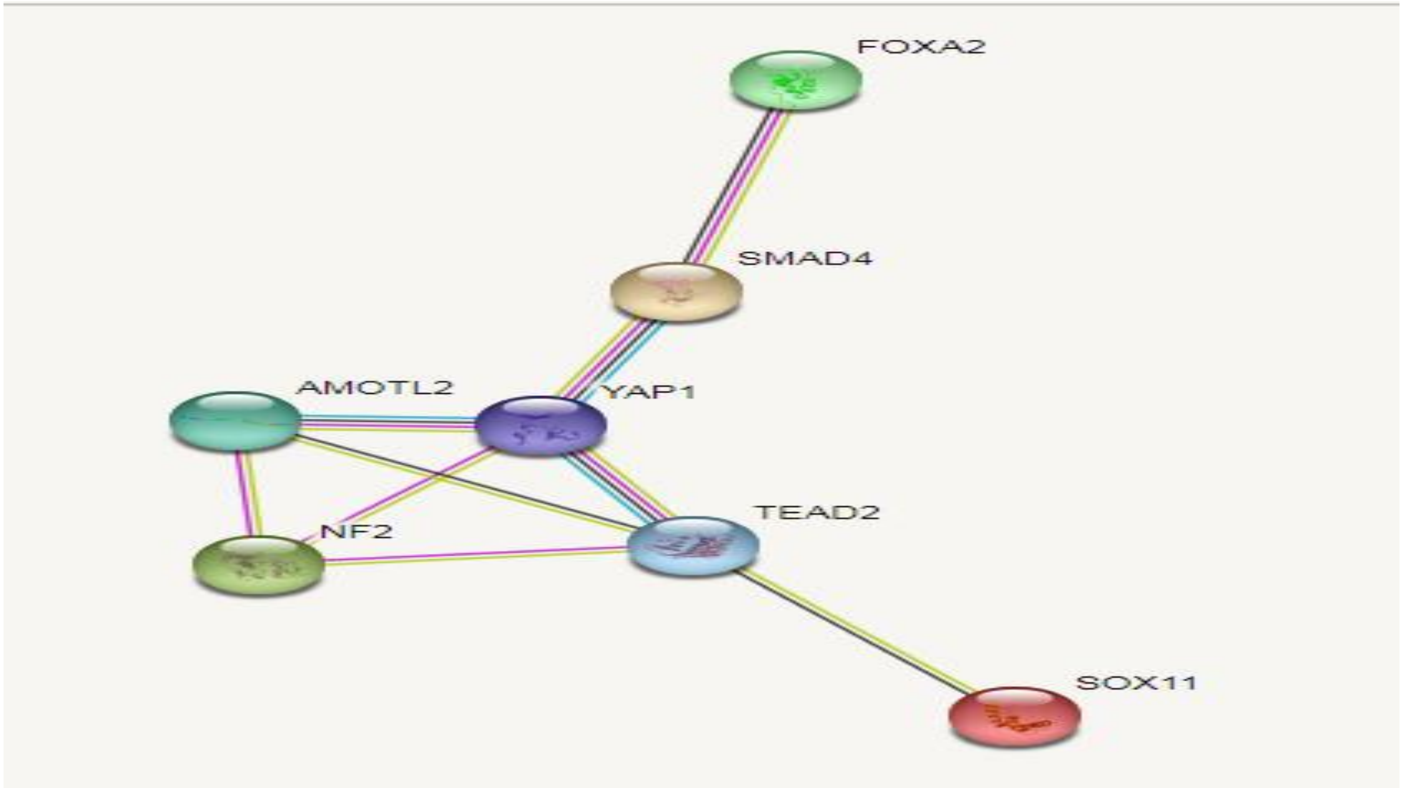

Figure S4A: print screen shows protein-protein interaction between the selected mRNAs and HIPPO target effectors by STRING database. https://string-db.org/cgi/network.pl?taskId=w75m42gDhIJ1

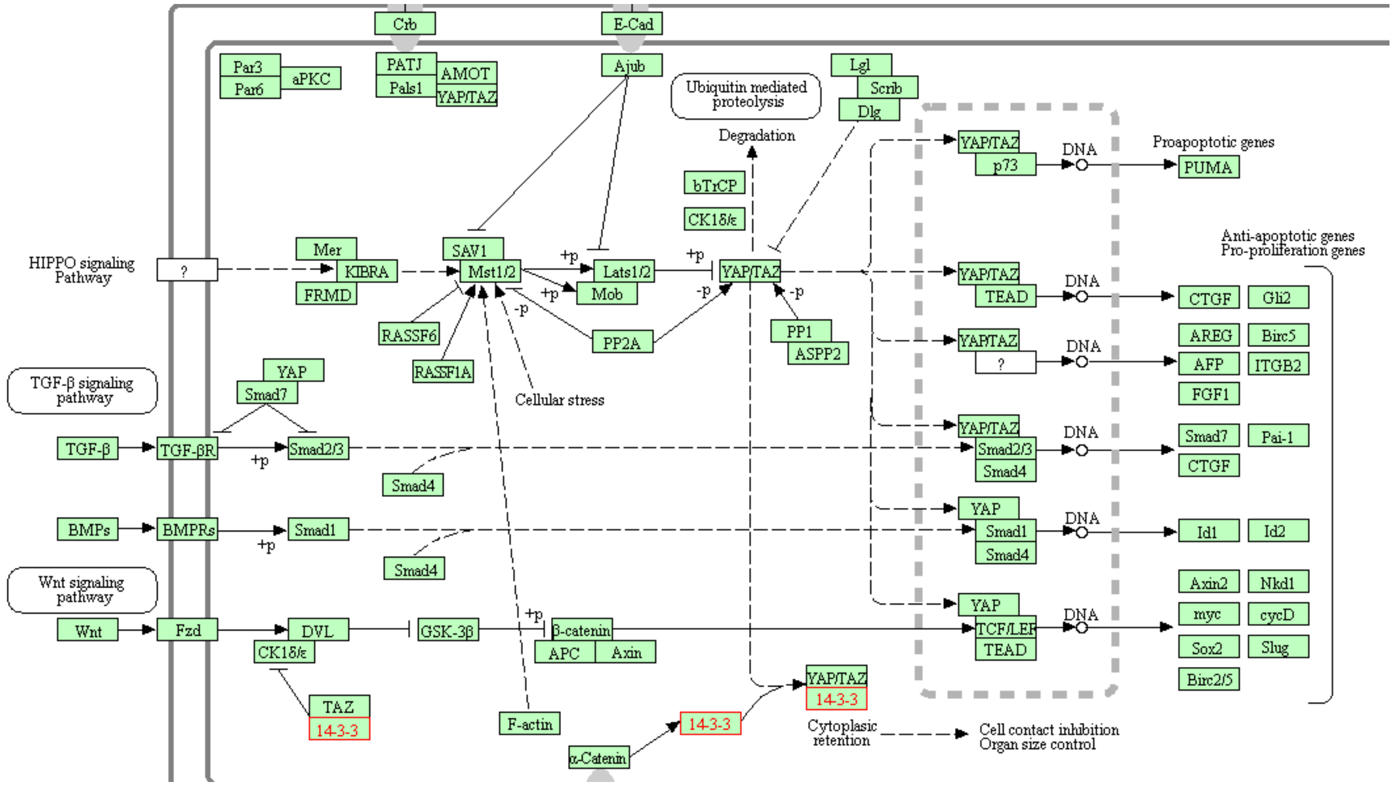

Figure S4B: print screen shows protein-protein interaction between the selected mRNAs and HIPPO target effectors by KEGG pathway of hippo.

https://www.kegg.jp/kegg-bin/show_pathway?select_scale=1.0&query=+YWHAB&map=hsa04390&scale=&orgs=&auto_image=&show_description=hide&multi_query=

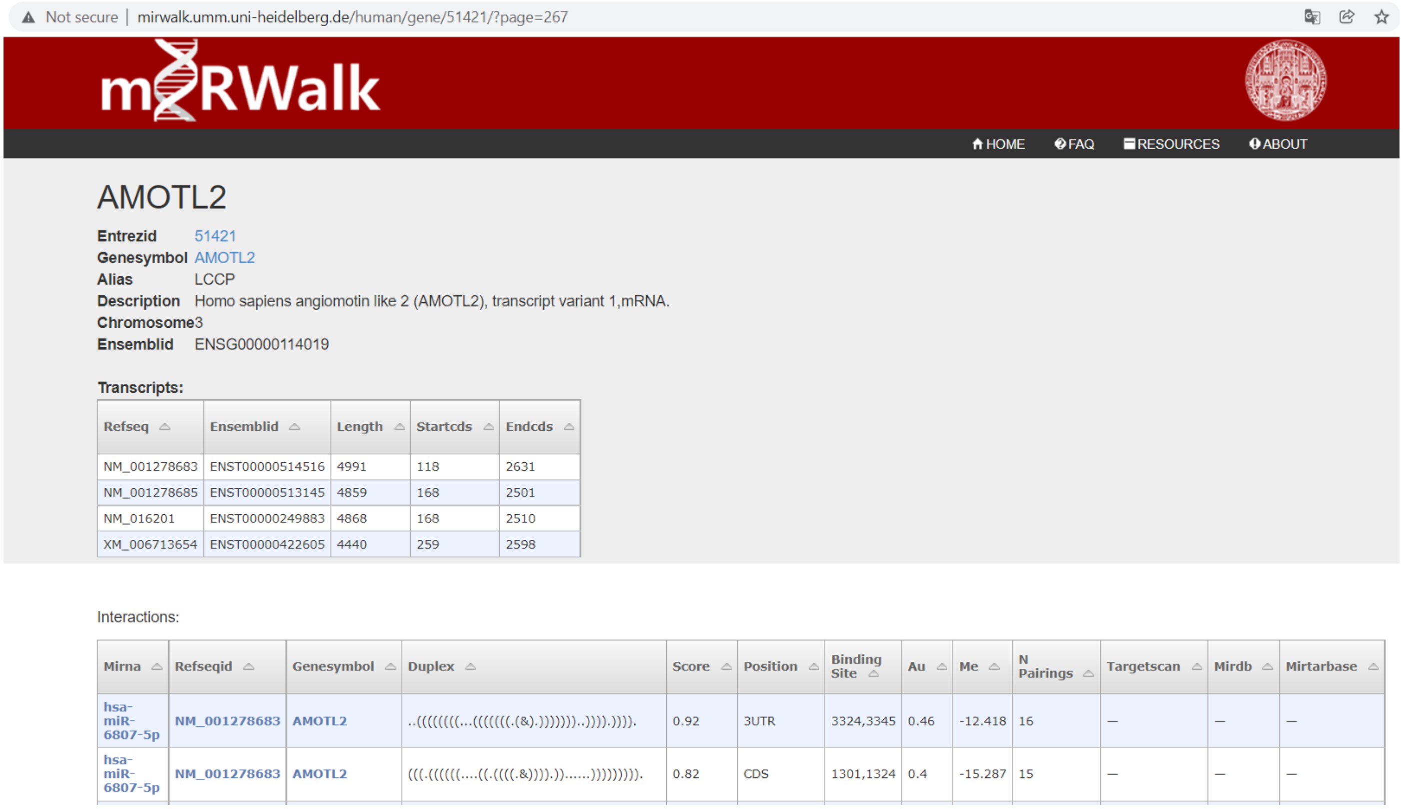
Not secure | mirwalk.umm.uni-heidelberg.de/human/gene/51421/?page=267

HOME FAQ RESOURCES ABOUT

AMOTL2

Entrezid 51421
Genesymbol AMOTL2
Alias LCCP
Description Homo sapiens angiomotin like 2 (AMOTL2), transcript variant 1,mRNA.
Chromosome 3
Ensemblid ENSG00000114019

Transcripts:

Refseq	Ensemblid	Length	Startcds	Endcds
NM_001278683	ENST00000514516	4991	118	2631
NM_001278685	ENST00000513145	4859	168	2501
NM_016201	ENST00000249883	4868	168	2510
XM_006713654	ENST00000422605	4440	259	2598

Interactions:

Mirna	Refseqid	Genesymbol	Duplex	Score	Position	Binding Site	Au	Me	N Pairings	Targetscan	Mirdb	Mirtarbase
hsa-miR-6807-5p	NM_001278683	AMOTL2	..((((((((...(((((((.(&).))))))..)))).)))).	0.92	3UTR	3324,3345	0.46	-12.418	16	—	—	—
hsa-miR-6807-5p	NM_001278683	AMOTL2	(((.((((((....((.((((.&))))).))......))))))))).	0.82	CDS	1301,1324	0.4	-15.287	15	—	—	—

Figure S5A:AMOTL2 mRNA is a direct target of miRNA-6807-5p that was retrieved from miRwalk database. It shows direct interaction between miRNA-6807-5p and AMOTL2 mRNA.

http://mirwalk.umm.uni-heidelberg.de/human/gene/51421/?page=267

SOX11

Entrezid 6664
Genesymbol SOX11
Alias CSS9;MRD27
Description Homo sapiens SRY-box transcription factor 11 (SOX11), mRNA.
Chromosome 2
Ensemblid ENSG00000176887

Transcripts:

Refseq	Ensemblid	Length	Startcds	Endcds
NM_003108	ENST00000322002	9002	339	1664

Interactions:

Mirna	Refseqid	Genesymbol	Duplex	Score	Position	Binding Site	Au	Me	N Pairings	Targetscan	Mirdb	Mirtarbase
hsa-miR-6806-3p	NM_003108	SOX11	((((((..((((((((((((...&)))))))))))))))))))).	1.00	3UTR	6953,6971	0.66	-7.795	17	—	—	—
hsa-miR-6806-3p	NM_003108	SOX11	....(((((((..(((((((..&)))))))..)))..)))).	0.92	CDS	528,547	0.46	-14.182	14	—	—	—
hsa-miR-6807-5p	NM_003108	SOX11	(((((.((((((.. ((((((((&)))))))).........................)))))))))).	0.92	CDS	1636,1683	0.43	-7.985	19	—	—	—
hsa-miR-6807-5p	NM_003108	SOX11	..((((((((((((((((((((&))))).)))))......)))))))))).	0.92	3UTR	7300,7329	0.66	-4.957	20	—	—	—

Figure S5B: SOX11 mRNA is a direct target of miRNA-6807-5p that was retrieved from miRwalk database. It shows direct interaction between miRNA-6807-5p and SOX11 mRNA. http://mirwalk.umm.uni-heidelberg.de/human/gene/6664/?page=204

SMAD4

Entrezid 4089
Genesymbol SMAD4
Alias DPC4;JIP;MADH4;MYHRS
Description Homo sapiens SMAD family member 4 (SMAD4), mRNA.
Chromosome 18
Ensemblid ENSG00000141646

Transcripts:

Refseq	Ensemblid	Length	Startcds	Endcds
NM_005359	ENST00000342988	8789	539	2197

Interactions:

Mirna	Refseqid	Genesymbol	Score	Position	Binding Site	Au	Me	N Pairings
hsa-miR-6806-5p	NM_005359	SMAD4	1.00	3UTR	4361,4394	0.38	-8.828	19
hsa-miR-6806-5p	NM_005359	SMAD4	0.85	3UTR	7430,7454	0.62	-11.09	19
hsa-miR-6806-3p	NM_005359	SMAD4	0.92	3UTR	2389,2415	0.6	-5.837	20
hsa-miR-6806-3p	NM_005359	SMAD4	0.85	3UTR	4316,4333	0.43	-12.371	15
hsa-miR-6806-3p	NM_005359	SMAD4	1.00	3UTR	8134,8156	0.59	-9.286	15
hsa-miR-6807-5p	NM_005359	SMAD4	0.92	5UTR	133,158	0.23	-10.876	17
hsa-miR-6807-3p	NM_005359	SMAD4	0.96	3UTR	4305,4330	0.46	-3.938	21

Figure S5C:SMAD4 mRNA is a direct target of miRNA-6807-5p that was retrieved from miRwalk database. It shows direct interaction between miRNA-6807-5p and SMAD4 Mrna.

http://mirwalk.umm.uni-heidelberg.de/human/gene/16399/?page=277

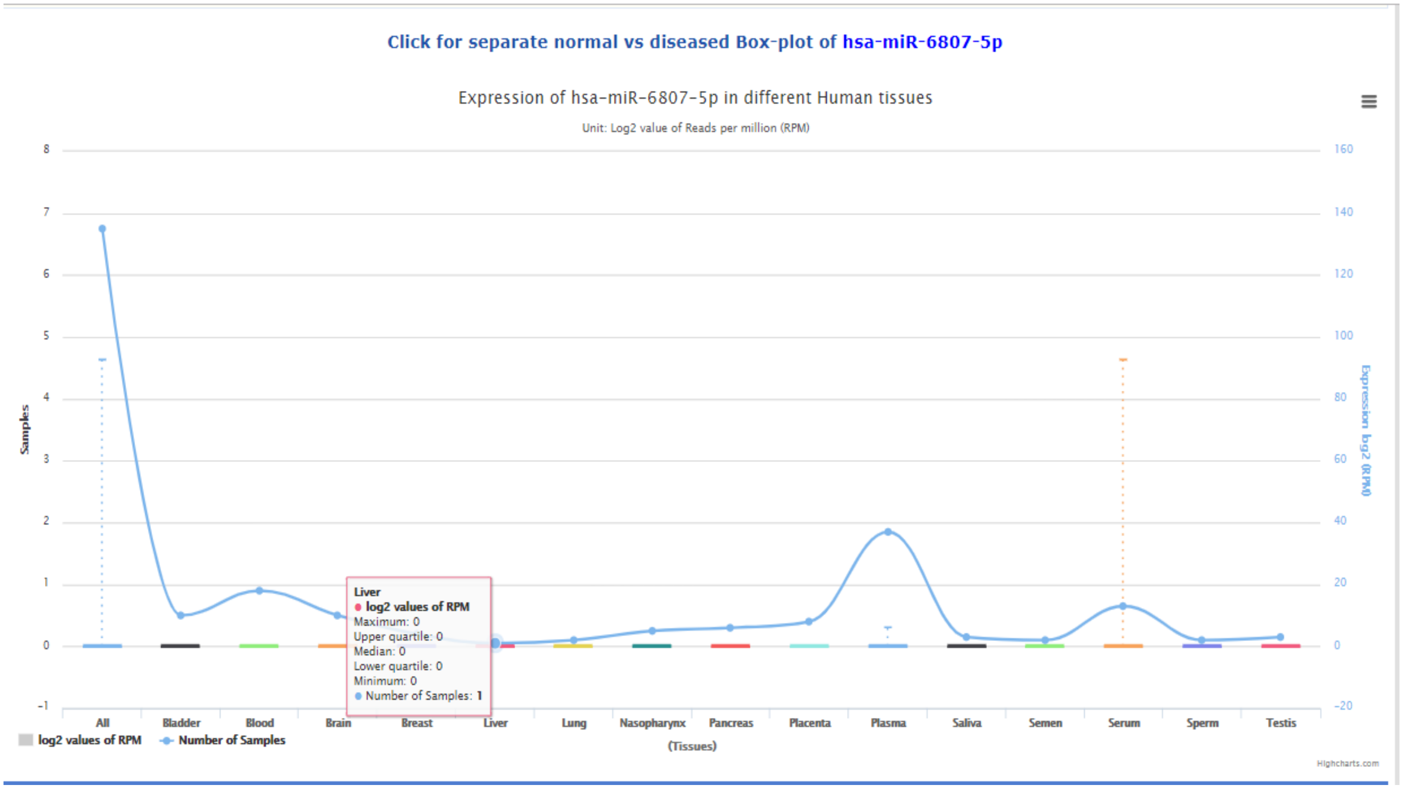

Figure S6A: Gene expression of has-miR-6807-5p in liver that was retrieved from miRmine database

http://guanlab.ccmb.med.umich.edu/mirmine/single.php?mirna=hsa-miR-6807-5p&tissue=liver&cline=

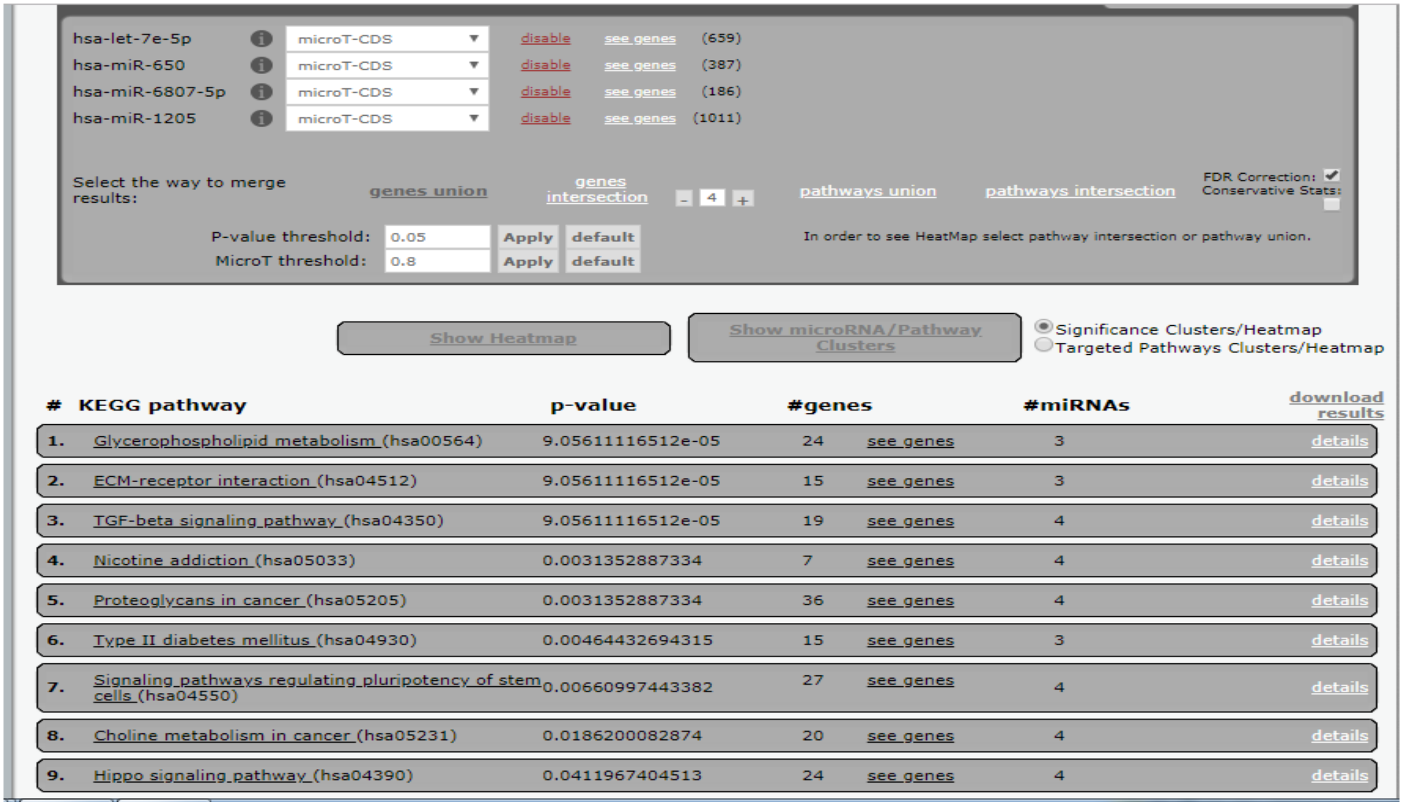

Figure S6B: Print screen shows pathway enrichment analysis of has-miR-6807-5p that was done by DIANA database tools: miRpath, http://snf-515788.vm.okeanos.grnet.gr/. It highlights the role of this miRNA in Hippo signaling.
